# Systemic administration of novel engineered AAV capsids facilitates enhanced transgene expression in the macaque CNS

**DOI:** 10.1016/j.medj.2022.11.002

**Published:** 2022-11-22

**Authors:** Alexandra C. Stanton, Kim A. Lagerborg, Liana Tellez, Allison Krunnfusz, Emily M. King, Simon Ye, Isaac H. Solomon, Mohammadsharif Tabebordbar, Pardis C. Sabeti

**Affiliations:** 1 Broad Institute of MIT and Harvard, Cambridge, MA, USA 02142; 2 Harvard Program in Virology, Harvard Medical School, Boston, MA, USA 02115; 3 Harvard Program in Biological and Biomedical Sciences, Harvard Medical School, Boston, MA, USA 02115; 4 Harvard-MIT Health Sciences and Technology, Massachusetts Institute of Technology, Cambridge, MA, USA 02142; 5 Harvard Medical School, Boston, MA, USA 02115; 6 Department of Pathology, Brigham and Women’s Hospital, Boston, MA, USA 02115; 7 Department of Organismic and Evolutionary Biology, FAS Center for Systems Biology, Harvard University, Cambridge, MA, USA 02138; 8 Lead Contact

## Abstract

**Background:**

Adeno-associated virus (AAV) vectors are a promising vehicle for noninvasive gene delivery to the central nervous system via intravenous infusion. However, naturally occurring serotypes have a limited ability to transduce the brain, and translating engineered capsids from mice to nonhuman primates has proved challenging.

**Methods:**

In this study, we use an mRNA-based directed evolution strategy in multiple strains of mice as well as in a *de novo* selection in cynomolgus macaques to identify families of engineered vectors with increased potency in the brain and decreased tropism for the liver.

**Findings:**

We compare the transgene expression capabilities of several engineered vectors and show that while some of our novel macaque-derived variants significantly outperform AAV9 in transducing the macaque brain following systemic administration, mouse-derived variants—both those identified in this study and those reported by other groups—universally did not.

**Conclusions:**

Together, this work introduces a class of primate-derived engineered AAV capsids with increased therapeutic potential and underscores the critical need for using appropriate animal models to both identify and evaluate novel AAVs intended for delivery to the human central nervous system.

**Funding:**

This work was funded primarily through an anonymous philanthropic gift to the P.C.S. lab at the Broad Institute of MIT and Harvard and by a grant from the Howard Hughes Medical Institute to P.C.S.

## eTOC blurb

Stanton *et al.* use a directed evolution strategy in mice and macaques to find AAV capsids with increased tropism for the brain after systemic administration. A resulting family of capsids show increased brain transduction and reduced tropism for the liver and other peripheral organs in juvenile macaques compared to AAV9.

## Graphical Abstract

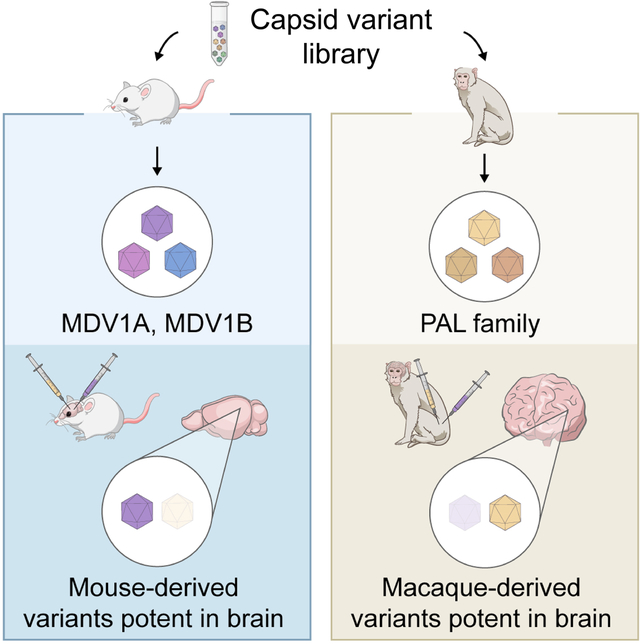

## Introduction

Adeno-associated virus (AAV) vectors are the vehicle of choice for gene therapy applications in the central nervous system (CNS) due to their low immunogenicity and ability to facilitate long-term gene expression in both dividing and non-dividing cells.^1–6^ Clinical and preclinical studies of recombinant AAV (rAAV)-based therapies with naturally occurring AAV serotypes have shown promise in the treatment of a variety of CNS disorders.^1,5,7–10^ However, the efficacy of rAAVs in transducing the CNS has been limited by the protective effect of the blood-brain barrier (BBB) and the broad tissue tropism of naturally occurring AAV serotypes, which together result in inefficient transduction of target cell populations in the CNS.^1,2,11^

Direct administration of rAAVs into the CNS via either the cerebrospinal fluid (CSF) or the brain parenchyma is a commonly employed and effective strategy to bypass the BBB, but has some notable drawbacks.^1,2,5,12–14^ Intraparenchymal injections require only small quantities of vector and are valuable for efficiently transducing focal cell populations.^5,13,15^ However, transduction of large or distal brain regions is restricted by limited vector diffusion, and axonal transport of rAAVs can only facilitate transduction of structures anatomically connected to the injection site.^13,16,17^ Moreover, the invasive nature of intracranial injections (which include both intraparenchymal as well as intracerebroventricular injections) risks surgical complication and has resulted in a high rate and severity of administration-related serious adverse events in human clinical trials.^14^ Intra-CSF injection, especially intrathecal injection into the cisterna magna or via a lumbar puncture, carries a lower safety risk during administration and results in broader vector distribution throughout the CNS, but tight junctions between ependymal cells limit vector penetration into deeper structures within the brain parenchyma.^2,14,18–20^

The discovery that the AAV9 serotype can cross the BBB has introduced the possibility of utilizing noninvasive systemic administration of rAAVs via the vascular system to facilitate widespread transduction across the CNS.^1,2,12,21^ Intravenous (IV) infusion has been employed in a number of clinical trials of CNS-targeted rAAV therapies^1,10^ and is the administration route of choice for an FDA-approved treatment for spinal muscular atrophy.^7^ However, systemic administration of naturally occurring AAV serotypes is complicated by sequestration of viral particles in the liver and the protective effect of the BBB, both of which limit rAAV bioavailability in the CNS.^1,2,11,22,23^ Achieving therapeutic efficacy in the CNS with systemic administration of rAAVs therefore requires large doses, sometimes exceeding 1E+14 vector genomes per kilogram body mass (vg/kg).^1,2,5,12^ In addition to posing significant manufacturing challenges, high dose rAAV therapy compounds the safety risk associated with an immune response in the liver, a phenomenon that has been observed in both clinical and preclinical studies.^1,2,22,24–27^

Engineering AAV capsids that display both enhanced transduction of the CNS and reduced transduction in peripheral organs following systemic administration will accelerate the development of CNS-targeted therapies with improved safety and efficacy at a reduced dose. Previous studies have successfully applied directed evolution techniques to generate novel AAV capsids with CNS-tropic properties *in vivo*,^28–32^ though translating these findings from mouse models to nonhuman primates (NHPs) has proved challenging and has complicated efforts to develop capsids with therapeutic potential in humans. A directed evolution strategy performed in Cre-transgenic C57BL/6J mice using the CREATE (Cre recombination-based AAV targeted evolution) method yielded potent CNS-tropic variants such as PHP.B and PHP.eB.^28,29^ However, the CNS-tropic properties of these variants translate poorly even to other mouse strains, and studies assessing IV administration of PHP.B in marmosets found that it failed to outperform AAV9 in CNS transduction.^33–36^ These findings cast doubt on the applicability of such vectors to human gene therapy and highlight the need to evaluate novel capsids in NHPs.

Recent studies have attempted to find less strain-specific CNS-tropic capsids using a multiplexed CREATE strategy in which directed evolution is performed across multiple mouse strains.^30^ Two PHP.eB-related variants identified in these efforts, AAV.CAP-B10 and AAV.CAP-B22, were later found to have improved CNS transduction in the marmoset brain compared to AAV9.^32^ Though variants demonstrating efficacy in marmosets likely hold greater therapeutic potential than those only capable of transducing the mouse brain, marmosets are much smaller and more evolutionarily distant from humans than are other common NHP models such as macaques. Given that positive results for certain engineered rAAVs in mice do not necessarily translate to NHPs^32,33,36^ and the extensibility of transduction data in marmosets to other NHPs is unknown, it is critical to assess the performance of novel rAAVs in appropriate animal models in order to identify candidate vectors for human gene therapy applications.

The unrealized potential of systemically administered rAAVs with CNS-tropic engineered capsids combined with the challenges in translating these capsids to NHPs serve as motivation for this work. In contrast to previous attempts to identify engineered capsids with therapeutic potential in the CNS, which typically involve selecting CNS-tropic capsids in mice, in this study we use an mRNA-based directed evolution strategy in both mice and cynomolgus macaques. The aim of this approach is to identify capsids that (i) retain CNS-tropic behavior across multiple animal models such that their properties may be conserved throughout the lineage; and (ii) have CNS-tropic behavior in NHP models that are the closest practical evolutionary neighbors to humans. In both cases, we aim to identify capsids with the highest degree of translational and therapeutic potential in humans.

## Results

### *In vivo* mRNA-based selection for CNS-tropic AAVs.

**Figure 1.**
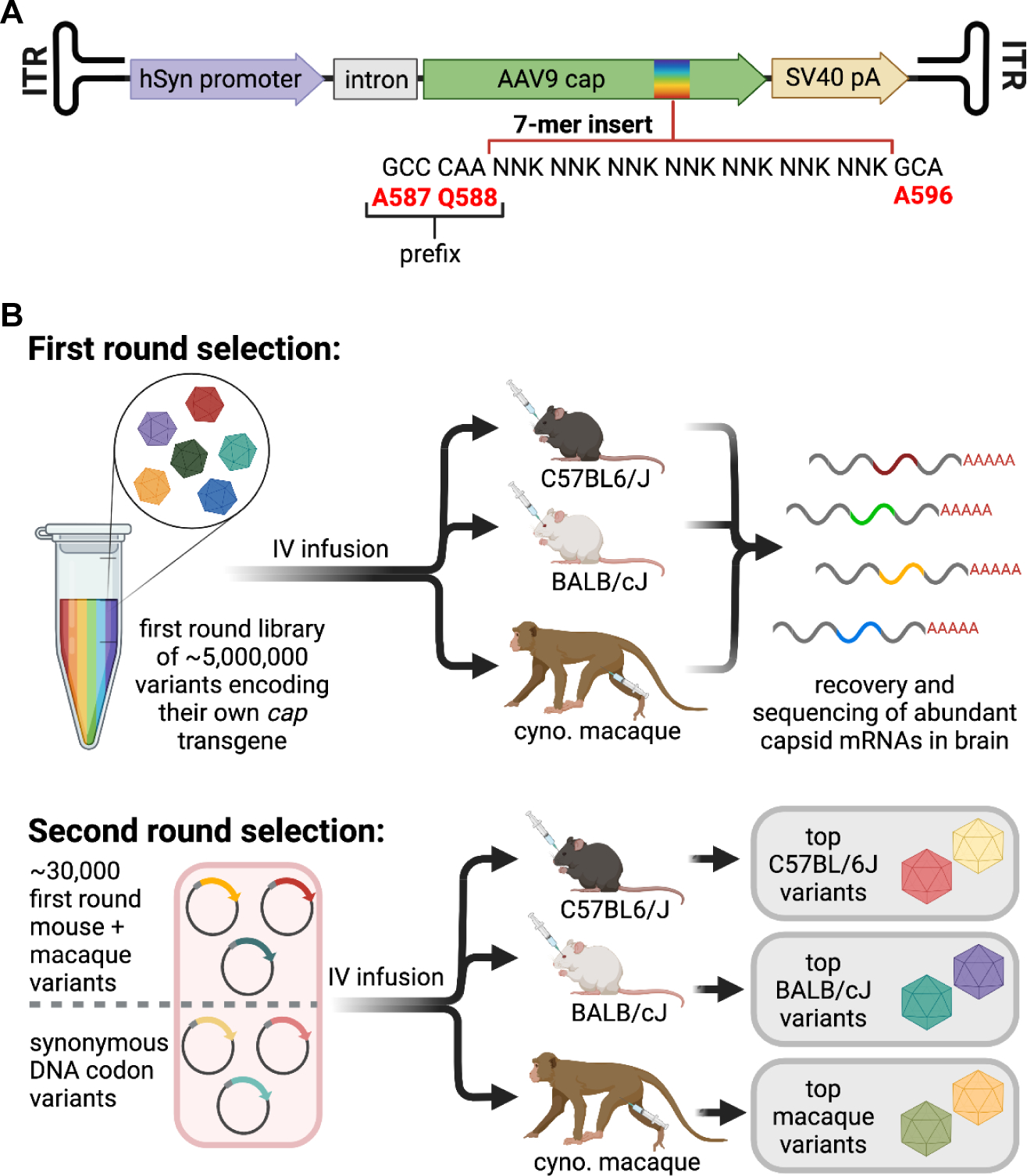
The DELIVER strategy selects for AAV capsid variants with an enhanced ability to transcribe transgene mRNA in the tissue of interest. **(A)** Map of self-packaging capsid library construct for DELIVER. **(B)** Schematic of selection using DELIVER. See also Table S1.

We developed AAV vectors with CNS-tropic properties in mice using the previously described *in vivo* directed evolution strategy DELIVER (**d**irected **e**volution of AAV capsids **l**everaging ***i****n* ***v****ivo* **e**xpression of transgene **R**NA).^37^ As the success of capsid variants in DELIVER is based on transgene mRNA expression, it preferentially selects for variants that are able to transcribe in addition to deliver genetic cargo. We first generated AAV9-based capsid libraries with a random 7-mer peptide inserted in the VR-VIII hypervariable region between residues Q588 and A589, a location known to permit exposure of the peptide on the capsid surface.^38,39^ The capsid library construct was flanked by inverted terminal repeats (ITRs), thereby eliciting self-packaging of the *cap* gene; that is, each capsid variant packages its own coding sequence as a transgene. To introduce selective pressure favoring capsid variants that preferentially transduce neurons, we placed the transgene under control of the neuron-specific human synapsin 1 promoter (hSyn) (Fig. 1A).

We performed two rounds of *in vivo* selection in parallel in C57BL/6J and BALB/cJ mice and in two-year-old cynomolgus macaques using expression of transgene mRNA as the selection criteria. We used 2 and 3 macaques in the first and second rounds of selection, respectively, as well as a total of 24 mice in each round. Following the first round of selection on a library of ≥5E+6 random 7-mer inserts, we generated a library for the second round of selection with the top 30,000 most enriched capsid variants in the brain, drawing 10,000 high-scoring variants from mice and 20,000 from macaques. We introduced a synonymous codon control where each of the 30,000 top peptides were encoded both by their experimentally recovered DNA sequence and by a synonymous DNA codon sequence (Fig. 1B). We also generated a complementary library where the two residues upstream of the 7-mer peptide insert were changed from AQ to DG, a modification thought to be responsible for the enhanced CNS-tropic properties of the engineered variant PHP.eB in mice.^29^

**Figure 2.**
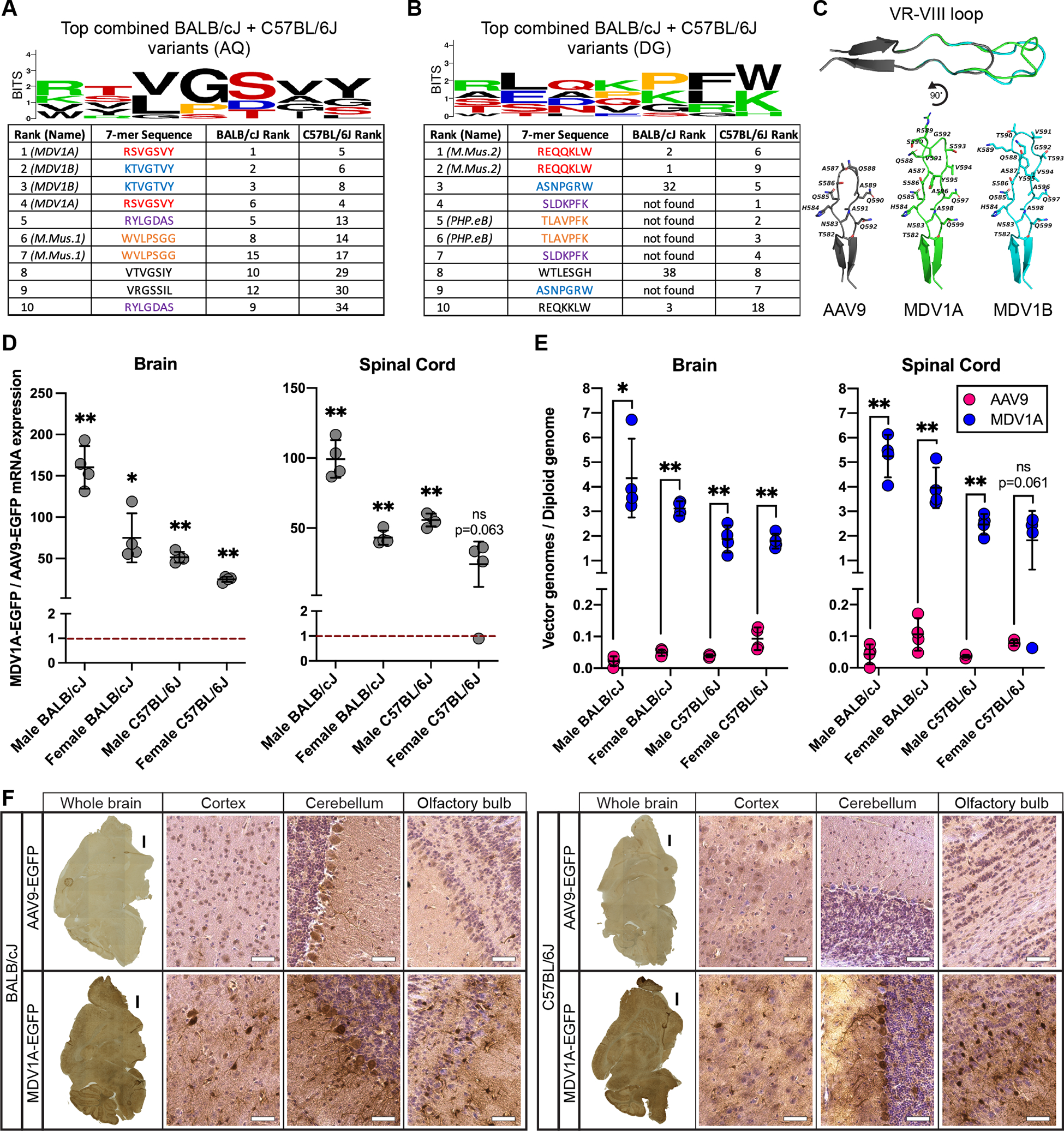
Selection with DELIVER yields potent CNS-tropic capsid variants in multiple mouse strains. **(A** and **B)** Amino acid sequence and logo of the 7-mer insert in the 10 most enriched capsid variants with the **(A)** AQ or **(B)** DG prefix in the brain of eight-week-old C57BL/6J and BALB/cJ mice injected with 1E+12 vector genomes (vg) virus library following two rounds of selection with DELIVER. Sequences with the same color in each table are encoded by synonymous DNA codons. **(C)** Predicted structure of the VR-VIII surface loops of AAV9, MDV1A, and MDV1B. **(D)** Fold difference in EGFP mRNA expression from MDV1A compared to AAV9 in the brain and spinal cord of male and female eight-week-old C57BL/6J and BALB/cJ mice injected with 1E+12 vg of MDV1A- or AAV9-CMV-EGFP. Dashed red line represents AAV9-CMV-EGFP expression normalized to 1. Data are represented as mean ± SD (n=3–4); *p<0.05, **p<0.01 (Welch’s *t*-test between MDV1A- and AAV9-injected mice with Holm-Šídák MCT). **(E)** Quantification of transgene delivery efficiency, expressed as vector genomes per diploid genome, of MDV1A- and AAV9-CMV-EGFP in the brain and spinal cord of eight-week-old C57BL/6J and BALB/cJ mice injected with 1E+12 vg of MDV1A- or AAV9-CMV-EGFP. Data are represented as mean ± SD (n=3–4); *p<0.05, **p<0.01 (Welch’s *t*-test between MDV1A- and AAV9-injected mice with Holm-Šídák MCT). **(F)** Representative images of 12 μm mouse brain sagittal cryosections immunostained for EGFP, from eight-week-old C57BL/6J and BALB/cJ mice injected with 5E+11 vg of MDV1A- or AAV9-CMV-EGFP. Insets show detailed features with hematoxylin nuclear counterstain (purple). Black scale bars: 1 mm; white scale bars: 50 μm. See also Table S1.

For the second round of selection in mice, we injected both the AQ and DG second-round libraries into separate sets of C57BL/6J and BALB/cJ mice. The identities of the most successful variants in mice differed depending on the prefix to the 7-mer insert (Fig. 2A,B and Table S1). Of the variants with the wild-type AQ prefix, the four most enriched DNA sequences averaged across both mouse strains—corresponding to two pairs of synonymous peptide sequences—encoded two highly similar variants. These two variants, *AQ*RSVGSVY and *AQ*KTVGTVY, are henceforth referred to as MDV1A and MDV1B (Mouse Double Valine), respectively (Fig. 2A), and are predicted to have similar but distinct secondary structure in the VR-VIII loop region (Fig. 2C). Of the variants with the modified PHP.eB-like DG prefix, the two most enriched DNA sequences are synonymous and encode the same peptide, *DG*REQQKLW (Fig. 2B). We recovered the sequences encoding PHP.B and PHP.eB from C57BL/6J but not BALB/cJ mice (Fig. 2B and Table S1), confirming that the CNS-tropic properties of PHP.B and PHP.eB are limited to C57BL/6J mice^33–36^ and further validating the DELIVER selection strategy.

We chose MDV1A for further characterization in mice based on its superior performance in both the C57BL/6J and BALB/cJ strains. We injected adult C57BL/6J and BALB/cJ mice of both sexes with 1E+12 vg of AAV9- or MDV1A-EGFP under control of the constitutive CMV promoter, which allowed us to capture transduction in non-neuronal cell types. Two weeks after administration of the vector, we assessed vector genome distribution and transgene expression in the brain and spinal cord of the 32 mice included in this experiment. MDV1A significantly outperformed AAV9 in both transgene delivery and expression in the brain of all groups of mice, demonstrating between a 25-fold and 160-fold improvement in transgene expression in the brain of female C57BL/6J and male BALB/cJ mice, respectively (Fig. 2D,E). In the spinal cord, MDV1A significantly outperformed AAV9 in transgene delivery and expression in three of the four groups of mice, with between a 43-fold and 99-fold improvement in transgene expression in female and male BALB/cJ mice, respectively (Fig. 2D,E). In the spinal cord of female C57BL/6J mice, there was a 24-fold performance difference between the two vectors that did not reach the threshold of statistical significance due to high variability in the data (Fig. 2D,E). Immunostaining of sagittal mouse brain sections revealed greater EGFP expression from MDV1A than AAV9, with relatively uniform distribution of EGFP throughout the brain (Fig. 2F).

**Figure 3.**
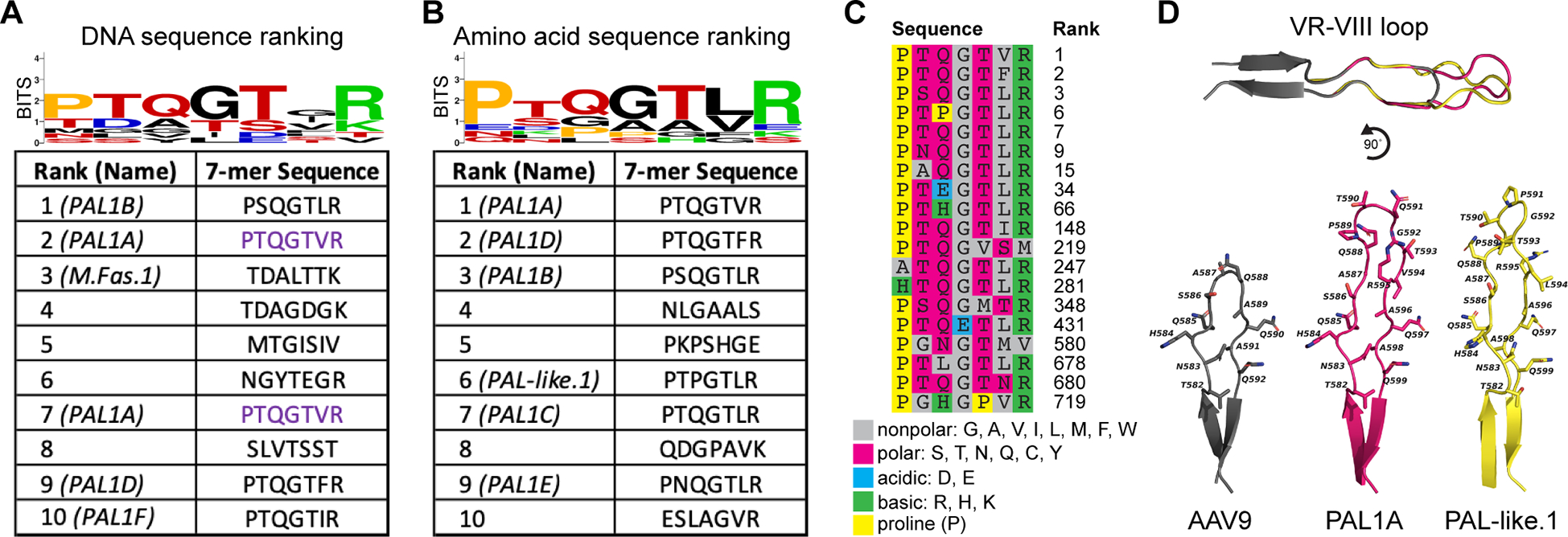
The Proline Arginine Loop (PAL) family of CNS-tropic capsid variants in cynomolgus macaques emerges after selection with DELIVER. **(A)** Amino acid sequence and corresponding peptide sequence logo of the 7-mer insert in the 10 most enriched DNA sequence-level capsid variants in the CNS of two-year-old cynomolgus macaques injected with 3E+13 vg/kg virus library following two rounds of selection with DELIVER. Sequences with the same color are encoded by synonymous DNA codons. **(B)** Amino acid sequence and logo of the 7-mer insert in the 10 most enriched amino acid-level capsid variants in the macaque CNS. The rank of each variant corresponds to the sum of the ranks of two synonymous DNA sequences. **(C)** Enriched variants contained in the PAL-like cluster generated by *k*-medoids clustering represented by the medoid sequence PTQGTLR. Individual residues are color-coded according to their functional properties to highlight conserved aspects of the sequence motif. The rank of each variant corresponds to the sum of the ranks of two synonymous DNA sequences. **(D)** Predicted structure of the VR-VIII surface loops of AAV9, PAL1A, and PAL-like.1. See also Figure S1 and Table S1.

In order to select for variants with CNS-tropic activity in primates, we also performed a second round of selection in three cynomolgus macaques. We used the same AQ library as in the second round of selection in mice, which included variants identified in the first round in both mice and macaques. We found that the variants most enriched in the macaque brain differed greatly from those identified in mice (Fig. 3A,B). Both with and without correcting for synonymous DNA sequences, the ten most enriched variants across the entire macaque CNS were dominated by a motif typified by a proline in position 1, the string QGT in positions 3–5, and an arginine in position 7. We also identified variants enriched in specific regions such as the cerebellum and spinal cord, though unlike in the CNS-wide results, these region-specific analyses did not converge on a single dominant motif (Fig. S1A,B).

We sought to identify sets of common CNS-tropic motifs more systematically by performing *k*-medoids clustering on the top 1000 macaque variants using a dissimilarity metric based on pairwise substitution scores between 7-mer peptides. The cluster represented by the medoid sequence PTQGTLR contained 19 variants, including 9 ranked in the top 100 sequences and 6 ranked in the top 10 (Fig. 3C). Many variants in this cluster—including most of the highest-performing variants—are broadly described by the motif PX_1_QGTX_2_R, where X_1_ is a polar uncharged residue and X_2_ is a nonpolar residue. We define the canonical **P**roline **A**rginine **L**oop (PAL) family of variants based on this motif, though more divergent PAL-like variants within the same cluster may share structural and functional properties with the canonical PAL variants. Computational modeling of the VR-VIII loop with the 7-mer insert predicted that canonical PAL variants share a nearly identical backbone conformation. However, even single-residue deviations from this core motif, such as the introduction of a proline at the third position in the sixth-ranked sequence PTPGTLR, may considerably alter the backbone conformation (Fig. 3D). *k*-medoids identified several additional clusters containing high-performing variants with conserved structural properties (Fig. S1C), but many variants were sorted into singleton clusters or small clusters with only two or three variants (Table S1).

### Benchmarking engineered AAV capsids in mice and macaques.

**Figure 4.**
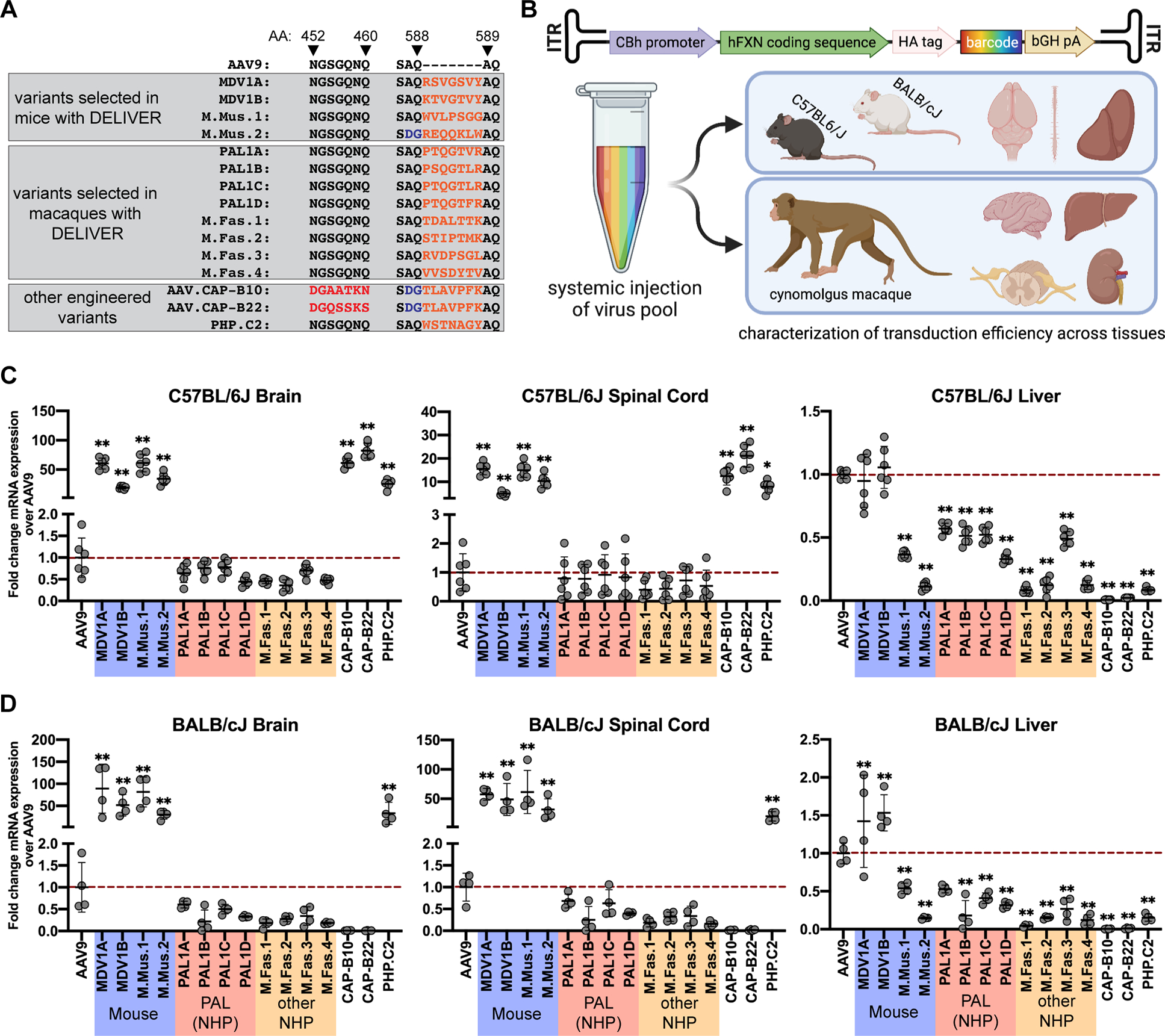
Mouse-derived variants outperform AAV9 in transduction of the mouse CNS. **(A)** Pool of capsid variants injected for characterization of the top mouse- and macaque-derived CNS-tropic variants. **(B)** Schematic of the barcoded human frataxin transgene and strategy for assessing the performance of top variants in cynomolgus macaques and C57BL/6J and BALB/cJ mice. **(C-D)** Fold difference in within-individual hFXN mRNA expression from different variants normalized to AAV9 in various tissues of **(C)** C57BL/6J mice and **(D)** BALB/cJ mice. Dashed red line represents AAV9-CBh-hFXN expression normalized to 1. Data are represented as mean ± SD (n=4–7 mice); *p<0.05, **p<0.01 (one-way ANOVA with Dunnett’s MCT and AAV9 as the control).

We assessed the relative performance of mouse- and macaque-derived engineered variants in order to determine if any variants had strong CNS-tropic properties in both mice and macaques. We performed a benchmarking experiment in C57BL/6J and BALB/cJ mice as well as in three juvenile cynomolgus macaques comparing four mouse-derived variants and eight macaque-derived variants from this study with AAV9. This panel also included three promising engineered variants developed by the Gradinaru lab using the M-CREATE platform: PHP.C2, which is known to transduce the CNS of both C57BL/6J and BALB/cJ mice,^30^ and AAV.CAP-B10 and AAV.CAP-B22, two PHP.eB-derived variants that were initially selected in Cre-transgenic mice but have demonstrated enhanced CNS-tropic activity in marmosets^32^ (Fig. 4A). The inclusion of these variants enabled us to directly compare them to our novel vectors without the confounding variables of dose, promoter, transgene, and other factors that could complicate a meta-study.

We generated rAAVs with each of these 16 capsids packaging human *frataxin* (hFXN)—the gene involved in the degenerative neurological disorder Freidreich’s ataxia—under control of the constitutive CBh promoter as a transgene. The CBh promoter was selected for its ability to induce ubiquitous long-term expression in the CNS and because its small size relative to the CBA promoter renders it attractive for therapeutic applications.^40^ Importantly, the use of a constitutive promoter allowed us to assess transduction in peripheral organs and tissues beyond the CNS. The transgene of rAAVs produced with each capsid contained a unique set of fifty 20-mer barcodes in the 3’UTR, which allowed us to associate sequenced hFXN transcripts with a specific capsid variant (Fig. 4B). We administered a pool containing equal proportions of each of these 16 capsid variants by IV injection to C57BL/6J and BALB/cJ mice and cynomolgus macaques at a total combined dose of 3E+13 vg/kg.

**Figure 5.**
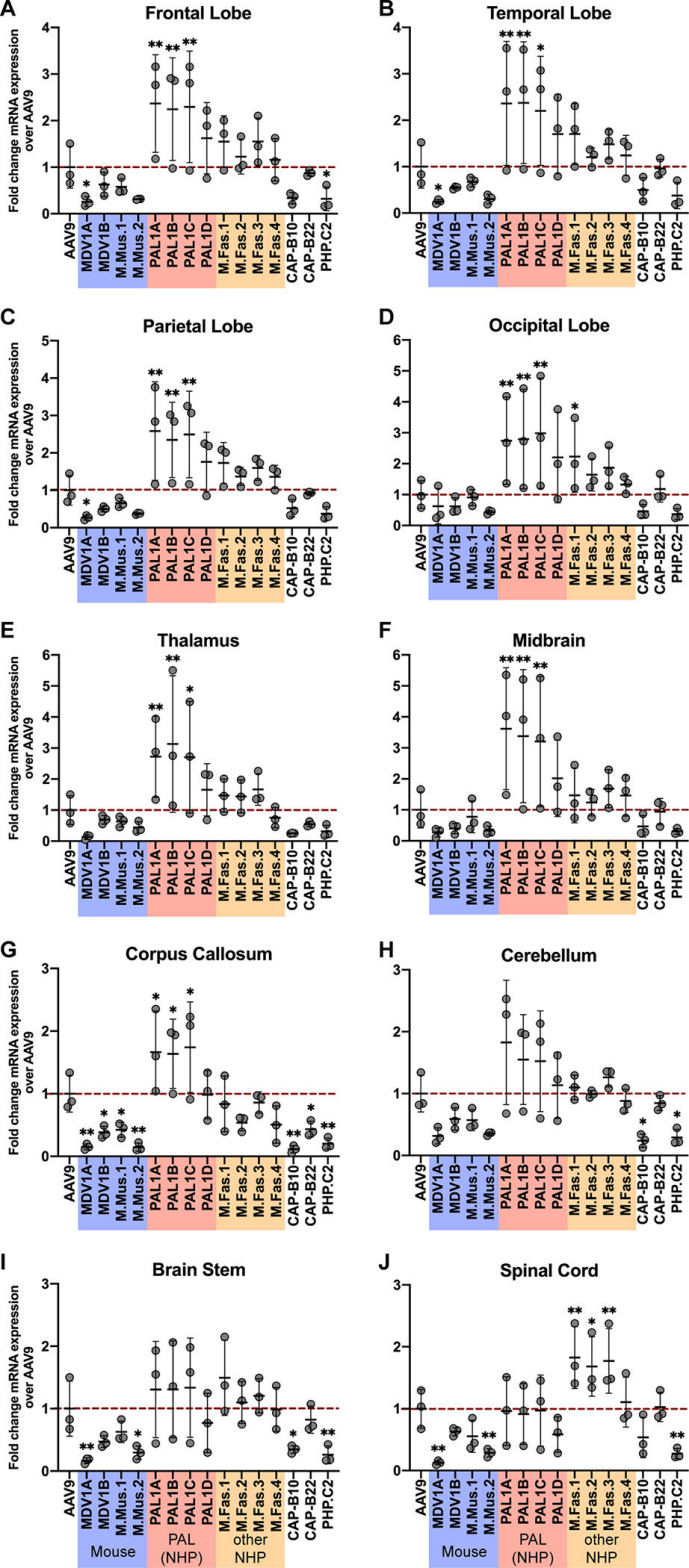
Macaque-derived variants outperform AAV9 and mouse- and marmoset-derived variants in transduction of the macaque central nervous system. **(A–J)** Fold difference in within-individual hFXN mRNA expression from different variants normalized to AAV9 in various regions of the cynomolgus macaque CNS. Dashed red line represents AAV9-CBh-hFXN expression normalized to 1. Data are represented as mean ± SD (n=3); *p<0.05, **p<0.01 (one-way ANOVA with Dunnett’s MCT and AAV9 as the control).

We found that the efficacy of each variant tested was linked to the animal model in which it was initially identified, and no variant exhibited cross-species CNS-tropic behavior. Quantification of hFXN mRNA expression revealed that none of the eight variants selected in macaques were capable of enhanced transduction of the brain or spinal cord of either mouse strain (Fig. 4C,D). Likewise, none of the seven mouse-derived variants that we tested effectively transduced the macaque CNS (Fig. 5). Remarkably, the AAV.CAP-B10 and AAV.CAP-B22 variants, which have previously been shown to outperform AAV9 in transducing the marmoset brain following systemic administration,^32^ did not show increased performance in any area of the macaque CNS (Fig. 5).

We were able to verify the efficacy of mouse-derived variants in the mouse strain in which each was initially discovered. All four mouse-derived variants identified in this study significantly outperformed AAV9 in transducing the brain and spinal cord of both mouse strains by a considerable margin, although of these four variants, only M.Mus.1 and M.Mus.2 were detargeted from the liver (Fig. 4C,D). The AAV.CAP-B10, AAV.CAP-B22, and PHP.C2 variants performed exceptionally well in the C57BL/6J brain and spinal cord (Fig. 4C). PHP.C2 was also able to successfully transduce the BALB/cJ brain and spinal cord in line with previous findings^30^ (Fig. 4D). However, as with the PHP.eB variant from which they were derived,^34,35^ AAV.CAP-B10 and AAV.CAP-B22 did not show tropism for the CNS of BALB/cJ mice (Fig. 4D).

**Figure 6.**
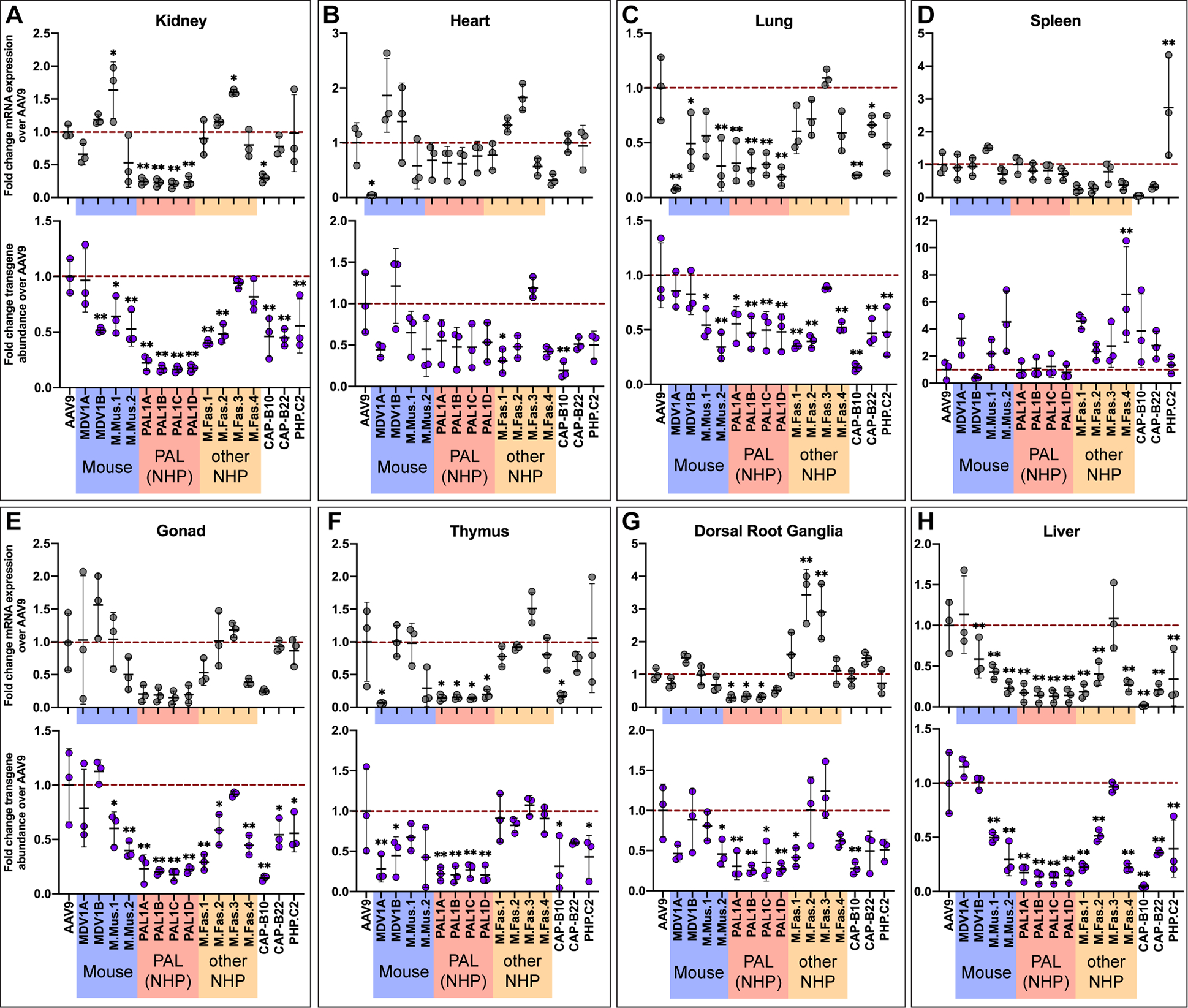
PAL variants are detargeted from several key peripheral organs in cynomolgus macaques. **(A-H)** Fold difference in within-individual hFXN mRNA expression (top panel of each frame) and vector genome abundance (bottom panel of each frame) from different variants normalized to AAV9 in cynomolgus macaque peripheral organs. Dashed red line represents AAV9-CBh-hFXN expression or vector genome abundance normalized to 1. Data are represented as mean ± SD (n=3); *p<0.05, **p<0.01 (one-way ANOVA with Dunnett’s MCT and AAV9 as the control).

We found that a number of variants discovered during the macaque selections in this study had increased potency over AAV9 in the macaque CNS. Three PAL family variants, PAL1A–PAL1C, were significantly better at transducing all four lobes of the macaque brain as well as the thalamus, midbrain, and corpus callosum, but not the cerebellum, brain stem, or spinal cord (Fig. 5). These three variants were additionally significantly detargeted from the dorsal root ganglia (DRG) (Fig. 6G). M.Fas.1–3 did not demonstrate significantly increased potency in the cerebrum, but unlike the PAL variants, they transduced the spinal cord more effectively than AAV9 (Fig. 5J). All macaque-derived variants except for M.Fas.3 were significantly detargeted from the macaque liver compared to AAV9 as measured by both transgene mRNA expression and vector genome delivery (Fig. 6H). All four of the PAL variants were additionally detargeted from the kidney, lung, and thymus by both metrics (Fig. 6A,C,F), and from the gonad by vector genome delivery but not transgene mRNA expression (Fig. 6E). These vectors showed comparable transduction levels to AAV9 in the heart and spleen (Fig. 6B,D).

### Individual characterization of a PAL variant.

We attempted to further optimize the PAL motif by performing a second-generation selection in two cynomolgus macaques with the PAL motif fixed, varying only the second and sixth position of the 7-mer insert as well as the three flanking residues immediately upstream of the insert. Modifications to this upstream flanking region, corresponding to SAQ in wild-type AAV9, have previously resulted in the enhanced transduction of PHP.eB compared to PHP.B in the C57BL/6J brain.^29^

From this selection we chose the second-generation PAL variant PAL2, with the sequence *EVG*PTQGTVR, for further study due to its relatively high performance and its similarity with the top first-generation variant PAL1A (*SAQ*PTQGTVR). We produced rAAVs with AAV9 and PAL2 each encoding hFXN under control of the CBh promoter and systemically administered 3E+13 vg/kg of each virus, for a total dose of 6E+13 vg/kg, to one female cynomolgus macaque referred to hereafter as macaque A. In order to distinguish between genomes and transcripts from the two different capsids, we tagged the hFXN transgene with an HA or FLAG epitope tag in PAL2 and AAV9 capsids, respectively. Constraints in funding and macaque supply amidst the COVID-19 pandemic limited the extent we could assess safety and performance of PAL2 and other PAL vectors in direct comparison with AAV9.

**Figure 7.**
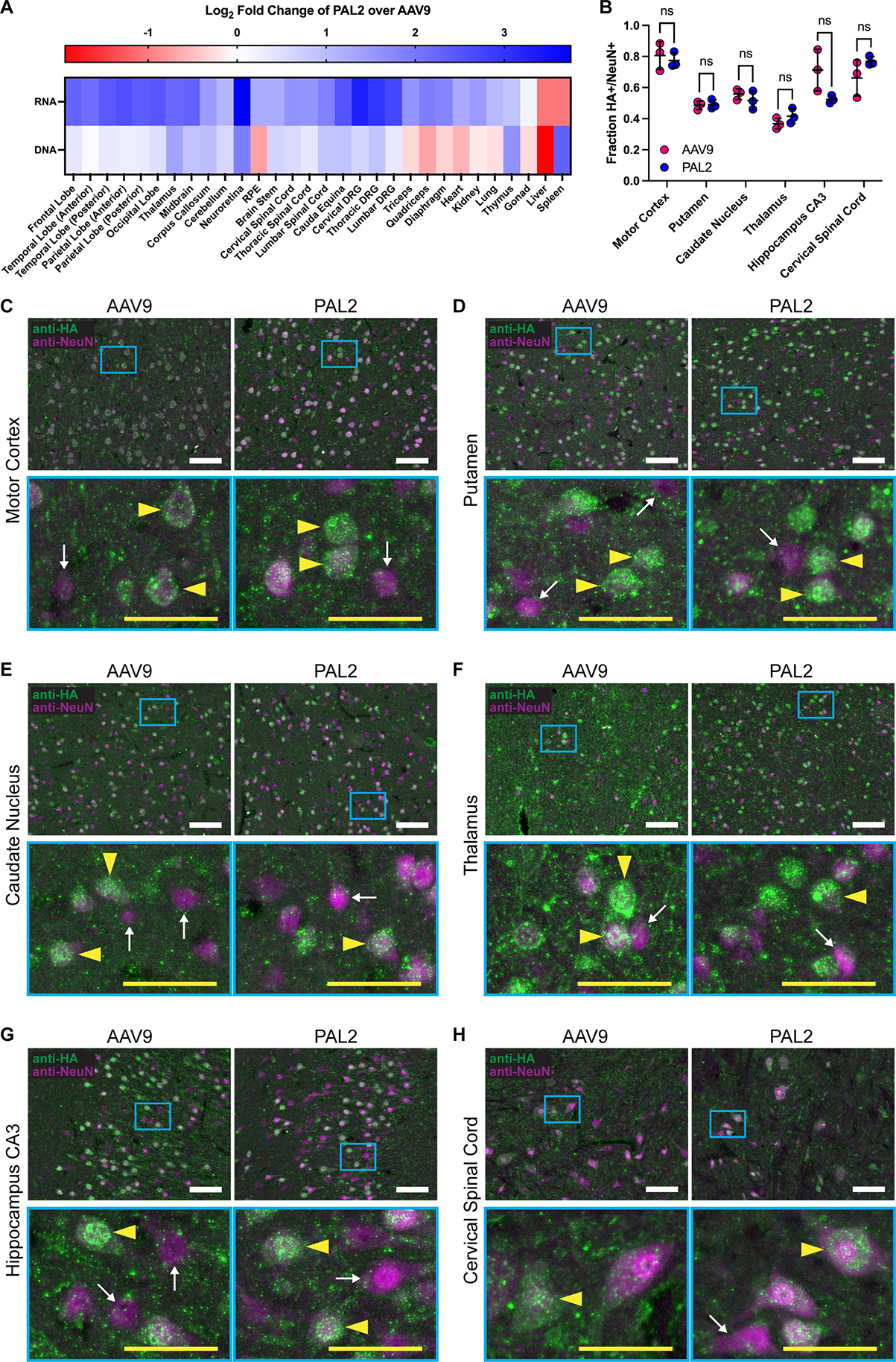
Second-generation capsid variant PAL2 transduces the macaque central nervous system in a head-to-head experiment with AAV9. **(A)** Heatmap of PAL2 transgene mRNA expression and vector genome abundance normalized to AAV9 in a single two-year-old female cynomolgus macaque three weeks after injection with 3E+13 vg/kg of each vector. Data are log_2_-transformed. **(B)** Fraction of NeuN+ neurons transduced by AAV9 or PAL2 delivering a CBh-hFXN-HA transgene as measured by immunostaining for the HA tag in conjunction with NeuN. In order to compare the same tag for both vectors, AAV9 and PAL2 data are derived from different two-year-old cynomolgus macaques injected with 3E+13 vg/kg of the vector in question; one male and one female, respectively. Data are represented as mean ± SD for n=3 replicate 12 μm cryosections and individual data points are the aggregate of three non-overlapping images of the same section taken with a 20X objective. Significance is determined by two-tailed Welch’s *t*-test with Holm-Šídák MCT. **(C-H)** Representative images depicting colocalization of hFXN-HA expression with respect to NeuN+ neurons in the macaque **(C)** motor cortex, **(D)** putamen, **(E)** caudate nucleus, **(F)** thalamus, **(G)** CA3 region of the hippocampus, and **(H)** ventral horn of the cervical spinal cord. Magnified frames (cyan) show detailed regions with representative HA+ transduced neurons (yellow arrowheads) and HA- untransduced neurons (white arrows). White scale bars: 100 μm; yellow scale bars: 50 μm. See also Figures S2–S5 and Tables S2–S3.

We assessed both vector transgene distribution and expression throughout a variety of tissues and found that PAL2 facilitated between a fourfold and sixfold increase in transgene mRNA expression throughout the cerebrum of macaque A compared to AAV9, except in the corpus callosum, where we only observed a 2.7-fold improvement (Fig. 7A and Table S2). As seen with the first-generation PAL1 variants, PAL2 transduction lagged in the cerebellum compared to the cerebrum, and in this experiment we found only a 2.1-fold increase in mRNA expression from PAL2 in the cerebellum (Fig. 7A and Table S2). Improvements in vector genome delivery were more modest; throughout the cerebrum and cerebellum we observed less than twofold more PAL2 vector genomes compared to AAV9 (Fig. 7A and Table S2).

We quantified the cell type tropism of AAV9 and PAL2 in the CNS by immunostaining for HA-tagged hFXN in macaque A and macaque B, a male cynomolgus macaque that was systemically injected with 3E+13 vg/kg of AAV9-CBh-hFXN-HA and 6E+13 vg/kg of PAL2-CBh-hFXN-FLAG (a total dose of 9E+13 vg/kg). Both vectors transduced a high fraction of neurons (measured by co-staining with NeuN) in all tested regions of the CNS, but neuronal transduction rates varied substantially between regions (Fig. 7B). Up to 80% of neurons in the motor cortex colocalized with HA signal, while this rate dropped to approximately 50% in the striatum (putamen and caudate nucleus) and 40% in the thalamus (Fig. 7B–H). Rates of astrocyte transduction (measured by co-staining with S100β) were comparably lower, except in the thalamus where 87% and 55% of astrocytes were transduced by AAV9 and PAL2, respectively (Fig. S2). The differences in transduction rates of either neurons or astrocytes between AAV9 and PAL2 were not statistically significant in any region tested (Fig. 7B and S2A).

As rAAVs have been successfully employed in the treatment of ocular diseases,^41^ we also assessed the relative efficiency of PAL2 in the retinal pigment epithelium (RPE) and neuroretina (retina absent the RPE). We found that PAL2 outperformed AAV9 in both transgene delivery and expression in the neuroretina by a factor of 3.8 and 13.4, respectively (Fig. 7A and Table S2). PAL2 vector genome abundance in the RPE was only 60% that of AAV9, but PAL2 nonetheless facilitated 2.3-fold greater mRNA expression in the RPE. For both AAV9 and PAL2, the hFXN-HA signal detected by immunostaining the neuroretina was highest in photoreceptor cells and in the outer plexiform layer where photoreceptors form synapses with horizontal cells (Fig. S3).

In line with our observations of first-generation PAL1 variants, PAL2 demonstrated one quarter of the vector genome abundance and one half of the mRNA expression in the liver relative to AAV9 (Fig. 7A and Table S2) in macaque A. Immunohistochemical staining the liver of macaque A for the HA and FLAG tags revealed a qualitatively lower amount of hFXN signal corresponding to PAL2 transduction compared to AAV9 (Fig. S4). Remarkably, this was also the case in macaque B despite the fact that this macaque received twice the dose of PAL2 compared to AAV9 (Fig. S4 and Table S3).

Though three first-generation PAL1 variants were significantly detargeted from the DRG (Fig. 6G), we found that PAL2 had increased DRG tropism compared to AAV9 (Fig. 7A and Table S2). Transduction of the DRG has been associated with neuroinflammation and neurodegeneration that can result in ataxia and other peripheral nervous system (PNS) deficits.^24,27,42–44^ We therefore assessed the spinal cord and DRG of macaque A for abnormal pathology, including mononuclear cell infiltration and neuronal cell body degeneration in the DRG and secondary axonopathy in the spinal cord. As this animal was administered a pool containing both AAV9 and PAL2, we are unable to distinguish the effects of one capsid from another; however, we were able to assess the combined effect of the two vectors in the context of this experiment. Multiple DRG and spinal cord segments from the cervical, thoracic, and lumbar regions of the spine were analyzed by a neuropathologist who established severity scores for each individual DRG and spinal cord segment ranging from 0 (within normal limits) to 5 as previously described.^43,45^ The macaque did not show abnormal pathology in any region tested (Fig. S5A,B). We additionally characterized the DRG and spinal cord pathology of macaque B, which received 1.5 times the total rAAV dose of macaque A. Like macaque A, macaque B did not show abnormal pathology in any region of the spinal cord (Fig. S5A), but we found that two of nine DRG assessed in that animal had minor lesions warranting a grade of 1 out of 5 (Fig. S5C). Both of the affected DRG originated from the lumbar spine; the one other lumbar DRG assessed from macaque B showed no abnormal pathology.

## Discussion

In this study we used the previously described DELIVER method^37^ to identify the novel PAL family of capsids that offer enhanced transduction in the CNS of juvenile cynomolgus macaques after a single dose IV infusion (Fig. 5 and 7, Table S2). These vectors were evolved *de novo* in macaques and demonstrate increased CNS tropism in macaques following systemic administration. We identified this family of capsids after just two rounds of selection, illustrating the utility of DELIVER in identifying potent AAV capsid variants in an additional tissue type. In a pooled characterization experiment assessing the performance of multiple engineered rAAVs in macaques, three PAL capsid variants (PAL1A–C) were capable of a moderate but highly statistically significant two- to threefold increase in transgene expression throughout the cerebrum (Fig. 5).

The second-generation variant PAL2 displayed an even greater four- to six-fold improvement in transgene mRNA expression in most areas of the cerebrum in an experiment with one macaque. PAL2 was also notably 13-fold more potent than AAV9 at transducing the neuroretina (Fig. 7A and Table S2), suggesting the feasibility of using a systemically administered rAAV to treat a disease affecting both the brain and retina, such as Krabbe disease. We did not observe a difference in the rates of transduction of either neurons or astrocytes in the CNS between AAV9 and PAL2 but found that both vectors transduced at least half of neurons and only a small fraction of astrocytes in all tested regions except the thalamus (Fig. 7 and S2). This stands in contrast to some earlier reports on the predominantly astrocytic tropism of IV-administered AAV9 in mice and NHPs,^21,46^ but other studies have also produced conflicting findings.^47,48^ We note that our results are drawn from only one animal subject for each vector, and the only other study utilizing hFXN-HA—which notably localizes to the mitochondria—as a transgene also reported low rates of astrocyte transduction by AAV9,^32^ suggesting that the transgene itself may affect observed levels of protein in different cell types.

In addition to demonstrating increased CNS tropism in macaques, the first-generation PAL variants PAL1A–C displayed a striking decrease in tropism for the macaque kidney, lung, thymus, and liver, both in terms of vector genome delivery and transgene mRNA expression (Fig. 6). Assessment of PAL2 transduction also indicated that the second-generation vector was detargeted from the liver (Fig. 7A and S4, Table S2). Identification of vectors with reduced liver tropism in particular is key to harnessing the advantages conferred by systemic administration, as sequestration of viral particles in the liver following IV infusion both decreases the effective dose at the target tissue and can lead to severe liver toxicity.^1,2,22,24–27^ These results therefore suggest that PAL vectors could achieve therapeutic efficacy following systemic administration at a reduced dose and with a lower risk of liver toxicity.

Three first-generation PAL variants also demonstrated reduced transduction in the DRG compared to AAV9 (Fig. 6G), though this effect was surprisingly inverted in the case of PAL2 (Fig. 7A and Table S2). DRG transduction has been associated with mononuclear cell infiltration, neuronal cell body degeneration, and secondary axonopathy that can result in ataxia and other PNS deficits.^24,43,44^ We assessed the spinal cord and DRG of two macaques injected with an rAAV cocktail of AAV9- and PAL2-CBh-hFXN. While we did not observe any histopathological signs of DRG toxicity or axonopathy in macaque A, we identified a few localized sites of inflammation in two of nine DRG in macaque B, which received a higher rAAV dose (Fig. S5). However, we note that the transgene identity and expression level, dose, and administration route all likely impact the degree of abnormal DRG pathology,^43,49^ and our findings are not necessarily extendable to other experimental designs.

We found that engineered variants identified in mice were universally unsuccessful at transducing the macaque CNS. Variants such as MDV1A that were selected in mice via DELIVER were able to potently transduce the CNS of two mouse strains (Fig. 2,4), but none of the four mouse-selected variants identified in this study outperformed AAV9 in transducing any area of the macaque CNS (Fig. 5). Even more surprisingly, AAV.CAP-B10 and AAV.CAP-B22, two variants that were selected in mice and shown to have enhanced CNS-tropic properties in marmosets,^30,32^ also failed to outperform AAV9 in transducing the CNS of cynomolgus macaques, a primate more closely related to humans. These results are strengthened by a concurring study that found AAV.CAP-B10 and AAV.CAP-B22 lack potency in the CNS of infant rhesus macaques.^50^

The failure of AAV transduction profiles to translate from mice to primates is well documented and has hampered development of CNS-targeted rAAV therapies,^33,36^ but this finding that the performance of some variants in one primate species may not translate even to another primate species has worrying implications for the field. From a preclinical standpoint, variants that retain their overall transduction behavior across a variety of model organism species, such as the MyoAAV class of variants identified in the skeletal muscle via DELIVER, are powerful tools.^37^ However, the results of this study suggest that the complexity of the CNS poses additional challenges in cross-species transduction and variant behavior is not necessarily conserved throughout the primate lineage. It is therefore of utmost importance that engineered AAVs are selected and evaluated in an appropriate animal model—one with the highest possible degree of similarity to humans—in order to maximize the likelihood of therapeutic efficacy in treating human neurological disorders.

Though they have lower translational potential than the PAL family, the vectors presented in this study with mouse-specific CNS tropism may have scientific value outside of the immediate context of clinical development. CNS-tropic AAVs with high potency in mice can be used for basic science research or early-stage preclinical studies using mouse models of genetic disease. For example, while PHP.B and PHP.eB are widely regarded to have a low potential for therapeutic use—at least in the context of systemic administration—they have proven useful in a variety of research applications. These two vectors have recently been used for purposes as diverse as gene therapy in human inner ear explants,^51^ delivering shRNA to elucidate the role of SPC25 in microglial outgrowth,^52^ and a novel method for retrograde tracing to study neural circuits.^53^ The properties of our mouse-specific CNS-tropic vectors, which have the added benefit over PHP.B and PHP.eB of being active in multiple mouse strains, may prove useful in similarly varied applications.

The mechanistic basis for BBB crossing and subsequent CNS tropism of novel capsids is a critically important area of research. AAV is commonly thought to cross the BBB via receptor-mediated transcytosis (RMT),^23,54,55^ and the PHP.B family of capsids are known to use LY6A as a receptor for RMT.^34,56^ The fact that the PAL vectors were only CNS-tropic in macaques and not in mice suggests that the RMT receptor for this family is either not present in mice or is poorly conserved between mice and primates. Identifying the mechanism used by PAL vectors to cross the BBB was not in the scope of this particular study, but research in this area could inform the design of next-generation capsids and guide future therapeutic applications.

We see the PAL family and other candidate vectors described here as a starting point for further investigation. Our study included 4 mouse experiments with a total of 75 mice across 2 strains, and 5 NHP experiments in a total of 12 juvenile cynomolgus macaques. This work encompassed multiple rounds of selection and directed evolution as well as limited characterization of one promising second-generation capsid. The properties of the PAL variants and other variants identified in this study may be further explored and enhanced in a number of ways. Firstly, additional iterations of directed evolution focusing on the 7-mer insert motif, flanking amino acids, or other areas of the capsid may result in improved or otherwise altered transduction properties as has been observed in the development of PHP.eB, AAV.CAP-B10, and AAV.CAP-B22.^29,32^ Secondly, while the advantages of systemic administration motivating this study are clear, refinement of intra-CSF delivery routes remains a promising area of research and may result in more robust transgene expression in the CNS^46^ at the possible expense of a higher risk of neuroinflammation and neurodegeneration.^42–44,57^ The combination of a PAL variant—or any other variant presented in this study—with an intra-CSF delivery method such as intracisternal injection^20^ may prove fruitful and suggest more varied applications for these vectors. Finally, the inclusion of tissue-specific microRNA targets on the vector transgene can reduce transgene expression and associated side effects in off-target tissues. Similar strategies utilizing microRNAs have shown promising results *in vivo* in the context of both liver and DRG detargeting.^45,58–61^

In summary, in this study we report the identification of a variety of AAV capsid variants with CNS-tropic properties in either mice or cynomolgus macaques, including a more extensively characterized family of variants containing a PAL motif that are capable of enhanced transduction of the macaque CNS and reduced sequestration in the liver following a single IV infusion. These results suggest that rAAV-based therapies with PAL variants may achieve therapeutic efficacy at a reduced dose, minimizing both safety concerns and vector manufacturing challenges. We additionally provide a list of the 1000 most highly enriched capsid variants in the CNS of macaques and two mouse strains (Table S1); further investigation and characterization of these variants may identify additional candidates for CNS gene therapy. Though we were unable to identify any variants able to potently transduce both the mouse and macaque CNS, this finding indicates a critical need for appropriate animal models and a move away from the current paradigm of evolving CNS-tropic AAVs in mice. It also suggests that further refinements to existing directed evolution strategies—including our own—may be necessary to identify a truly cross-species compatible CNS-tropic capsid. This study, and particularly our characterization of the PAL family of variants in macaques, opens up new avenues of inquiry and development towards safe and effective rAAV therapies for diseases of the CNS in humans.

### Limitations of Study

This study was broadly limited by the cost and labor required for NHP experiments and the availability of suitable macaques, which was impacted by the COVID-19 pandemic. All NHP experiments presented in this study therefore include only two or three subjects each, for a total of twelve subjects across five experiments. In our experiment characterizing first-generation PAL variants alongside other variants in three macaques (Fig 4–6), we note that the macaque with the lowest level of efficacy from the PAL variants was the only female included in the experiment. Sex has previously been shown to affect hepatic rAAV gene transfer in mice;^62^ it is unknown if there are sex-specific differences in transduction in other tissues or in primates. Our findings on the cell type tropism of PAL2 and AAV9 (Fig. 7 and S2) are based on extremely limited data; it remains plausible that these findings are an artifact of the staining procedure, as the high cost of healthy macaque brain and spinal cord tissue from commercial sources precluded our use of an HA-negative control. Additional studies with a greater number of NHP subjects will be required to fully assess the safety and performance of the PAL variants and other variants identified in this work.

## STAR Methods

### Resource Availability

#### Lead Contact

Requests for further information, resources, and reagents should be directed to and will be fulfilled by the lead contact, Alexandra Stanton (acstanton@g.harvard.edu).

#### Materials Availability

Reagents generated in this study will be made available on request, considering the terms of materials transfer agreement (MTA) for the modified reagents. We may require a completed MTA if there is potential for commercial application.

#### Data and Code Availability

- Next generation sequencing data have been deposited at NCBI Sequence Read Archive (SRA) with the bioproject ID PRJNA895493 and are publicly available as of the date of publication. The accession number is also listed in the key resources table.
- All original code has been deposited at Zenodo with the DOI 10.5281/zenodo.7262232 and is publicly available as of the date of publication. The DOI is also listed in the key resources table.
- Any additional information required to reanalyze the data reported in this paper is available from the lead contact upon request.

### Experimental Model and Subject Details

All animal care, housing, and experimental procedures were carried out in accordance with the Broad Institute’s Institutional Animal Care and Use Committee (IACUC) and Biomere’s IACUC.

#### Mice

Eight-week-old healthy and treatment-naive male and female C57BL/6J (JAX, #000664) and BALB/cJ (JAX, #000651) mice were purchased from the Jackson laboratory. Mice were housed in standard conditions in accordance with the Broad Institute’s IACUC. All mouse AAV injections were performed retro-orbitally, which has been shown to be faster and more humane than tail vein injection while the two methods are kinetically equivalent in the general case of IV administration of drugs or other compounds.^63–65^ Tissue samples were collected from the mice after whole body perfusion with either Dulbecco’s phosphate-buffered saline (DPBS) or DPBS followed by 4% paraformaldehyde (PFA).

#### Cynomolgus macaques

Non-human primate studies were performed at Biomere (Worcester, MA, USA) in accordance with their standard operating protocols and procedures approved by their IACUC. Healthy and treatment-naive male and female cynomolgus macaques, approximately two years of age, were sourced from Alpha Genesis and housed in standard conditions at Biomere. Only animals with a serum AAV9 neutralizing antibody titer of less than 1:3 were selected for *in vivo* studies. This cutoff was chosen conservatively in response to studies suggesting that a neutralizing antibody titer of between 1:5 and 1:10 for AAV8 in NHPs and 1:4 for AAV9 in rodents and non-primate large animal models was sufficient to inhibit rAAV transduction.^66–68^ Macaques were injected via an IV bolus injection for all experiments. Animals were euthanized and perfused with DPBS, after which CNS, muscle, and organ tissues were harvested. Tissue samples were preserved in RNAlater stabilization solution (Thermo Fisher) prior to downstream processing.

#### Cell lines

HEK293 cells used for rAAV and AAV library production were purchased from ATCC (ATCC, CRL-1573). Cells were maintained in DMEM supplemented with 5% v/v fetal bovine serum at 37°C and 5% CO_2_.

### Method Details

#### Constructs

CMV-*EGFP* plasmids used to produce EGFP-encoding AAV9 and MDV1A were generated by cloning the cytomegalovirus (CMV) promoter, EGFP coding sequence, and bovine growth hormone polyadenylation signal (bGH pA) into the pZac2.1 construct purchased from the University of Pennsylvania vector core. The AAV capsid library recipient plasmid was generated by assembling the human synapsin 1 (hSyn) promoter, AAV2 rep P40 promoter fragment, AAV9 *cap*, and SV40 polyadenylation signal into an ITR-containing backbone. The AAV2 P40 promoter permits Rep-dependent expression of *cap* in HEK293 cells where Rep is provided *in trans* for the purpose of vector production, while the hSyn promoter drives *cap* expression in neurons *in vivo.* The AAV9 *cap* gene on the library recipient plasmid was modified to contain BsmBI restriction sites immediately after Q486 and Q588 to facilitate insertion of a variable peptide sequence. The pZac2.1-CBh-hFXN-HA-bGH and pZac2.1-CBh-hFXN-FLAG-bGH plasmids were assembled by cloning the hybrid CBh promoter,^40^ human frataxin coding sequence, HA tag, and bGH pA into the pZac2.1 plasmid backbone between the ITRs. As previously described, for the pooled characterization experiment, 12 bp barcodes were inserted immediately after the HA tag in the pZac2.1-CBh-hFXN-HA-bGH plasmid.^37^ Each variant in the pooled characterization experiment was associated with 50 unique barcodes that were randomly generated with a minimum Hamming distance of four between any two barcodes.

First round AAV capsid library plasmids were prepared by amplifying a section of the AAV9 *cap* gene with an NNK degenerate reverse primer to produce fragments encoding every possible random 7-mer peptide insertion after Q588. These fragments were then introduced into the BsmBI-digested capsid library recipient plasmid. This library has a theoretical diversity of 20^7^ (1.28E+9) variants at the amino acid level, and we were able to identify at least 5E+6 unique capsid variants in our first-round capsid libraries based on next-generation sequencing. Second round libraries were generated through a similar method, but instead of NNK degenerate primers, a synthetic oligo pool (Agilent) was used to produce only selected variants of interest and synonymous DNA codon replicates. Libraries with the fixed PAL motif X_1_X_2_X_3_PX_4_QGTX_5_R were generated with a reverse primer containing NNK degenerate codons at the variable positions X_1_-X_5_. All cloning was performed using the NEBuilder HiFi DNA assembly master mix (New England Biolabs).

#### Capsid library and recombinant AAV production

AAV capsid libraries and rAAVs were produced in HEK293 cells with the usual triple-plasmid transfection method.^69^ Briefly, HEK293 cells were seeded into 15 cm dishes at a density of 2E+7 cells per dish and transfected the following day using PEI MAX (Polysciences). For individual rAAV production, cells were transfected with 16 μg pALDX-80 (Aldevron), 8 μg Rep2/Cap plasmid, and 8 μg of the ITR-containing transgene plasmid per dish. rAAVs were harvested from the cells and media and purified by ultracentrifugation over an iodixanol gradient as previously described.^69^ A slightly modified protocol was used for the production of AAV capsid libraries. Firstly, only 10 ng of the AAV capsid plasmid library was used per dish in order to prevent cross-packaging of variants and the formation of mosaic capsids, and 8 μg of pUC19 plasmid was included in the transfection to maintain the total amount of transfected plasmid as previously described.^28^ Secondly, 8 μg of Rep-AAP plasmid (a generous gift from Benjamin Deverman)^28^ was used in place of the Rep2/Cap plasmid. Finally, virus was harvested after 60 hours rather than the usual 120 hours in order to limit secondary transduction of virus-producing cells. All AAVs were titered by qPCR.

#### In vivo selection in mice and cynomolgus macaques

First- and second-round selections were performed in eight-week-old C57BL/6J and BALB/cJ mice and in two-year-old macaques. Six male and six female mice from each strain were used for each selection, and each mouse received a 1E+12 vg injected dose of either the AQ or DG capsid variant library. For the first round of selection in macaques, one male and one female were injected with 1E+13 vg/kg capsid library. For the second round of selection in macaques, two males and one female were injected with 3E+13 vg/kg AQ capsid library. For selection on the fixed PAL motif with modified flanking amino acids in macaques, two males were injected with 3E+13 vg/kg. In all selection experiments, three weeks after injection, animals were euthanized by perfusion with saline and whole brains were harvested. Spinal cords were additionally harvested from macaques. Fresh tissues were cut into 2 mm^3^ cubes and snap-frozen in liquid nitrogen before being stored at −80°C. Total RNA was extracted from at least 80% of the total tissue volume with TRIzol (Thermo Fisher) and mRNA was enriched from total RNA samples with oligo dT beads (New England Biolabs) and treated with Turbo DNase (Thermo Fisher). Subsequently, cDNA was synthesized with SuperScript IV reverse transcriptase (Thermo Fisher) and a capsid-specific primer (5’-GAAAGTTGCCGTCCGTGTGAGG-3’). Capsid variant sequences were then amplified with Q5 High-Fidelity 2X master mix (New England Biolabs) and primers flanking the 7-mer insert (5’-ACAAGTGGCCACAAACCACCA-3’ and 5’-GGTTTTGAACCCAGCCGGTC-3’) that added Illumina adaptors and unique indices (New England Biolabs). Amplicons were pooled at an equimolar ratio and sequenced on an Illumina NextSeq.

#### In vivo rAAV characterization

For comparison of vector genome delivery and transgene mRNA expression between AAV9 and MDV1A in mice, four male and four female eight-week-old C57BL/6J mice and four male and four female eight-week-old BALB/cJ mice were injected with 1E+12 vg of AAV9- or MDV1A-CMV-EGFP. Tissues were harvested two weeks after injection. For comparison of transgene expression via immunostaining, eight-week-old C57BL/6J and BALB/cJ mice were injected with 5E+11 vg of AAV9- or MDV1A-CMV-EGFP. Tissues were again harvested two weeks after injection. For comparison of PAL2 and AAV9, one female macaque was injected with 3E+13 vg/kg each of AAV9-CBh-hFXN-FLAG and PAL2-CBh-hFXN-HA. One male macaque was additionally injected with 3E+13 vg/kg of AAV9-CBh-hFXN-HA and 6E+13 vg/kg of PAL2-CBh-hFXN-FLAG. These macaques were euthanized by saline perfusion and tissues were harvested three weeks after injection.

For the pooled rAAV characterization experiment (Fig. 4–6), eight in-house macaque-derived capsids, four in-house mouse-derived capsids, AAV.CAP-B10, AAV.CAP-B22, PHP.C2, and AAV9 were used to produce rAAVs packaging the barcoded CBh-hFXN-HA-bGH transgene. Equal amounts of each of the 16 barcoded rAAV pools were mixed and injected into two male and one female two-year-old macaques, three male and four female eight-week-old C57BL/6J mice, and two male and two female eight-week-old BALB/cJ mice. All animals were injected with a combined dose of 3E+13 vg/kg, or 1.875E+12 vg/kg per capsid variant. Animals were euthanized by saline perfusion and tissues were harvested four weeks after injection and total RNA was extracted and treated as described above, and macaque liver DNA was additionally isolated with QuickExtract DNA extract solution (Lucigen). cDNA was synthesized with a bGH pA-specific primer (5’- TTCACTGCATTCTAGTTGTGGTTTG-3’) and DNA and cDNA were amplified with Q5 High-Fidelity 2X master mix and primers flanking the barcode region (5’-CCATACGATGTTCCAGATTACGC-3’ and 5’-CAATGTATCTTATCATGTCTGCTCGA-3’). Amplicons with Illumina adapters and unique indices were pooled at equimolar ratios and sequenced on an Illumina NextSeq.

#### Transgene delivery and expression quantification

For transgene expression quantification, RNA was extracted from mouse and macaque tissues with TRIzol (Thermo Fisher) and treated with Turbo DNase (Thermo Fisher). cDNA was synthesized with SuperScript IV reverse transcriptase (Thermo Fisher) with an oligo-dT primer. For transgene delivery (vector genome quantification) experiments, DNA was extracted from mouse and macaque tissues with QuickExtract DNA extract solution (Lucigen) following pulverization of snap-frozen tissue with a Geno/Grinder 2010 (SPEX SamplePrep). Transgene mRNA and DNA were measured by qPCR using Taqman assays specific to the transgene (EGFP or HA- or FLAG-tagged hFXN) mRNA or DNA or a housekeeping control (GAPDH). All measurements were quantified based on a standard curve generated by amplifying a gblock containing the target sequence of each Taqman assay, and absolute quantities of transgene mRNA and DNA were then normalized to the housekeeping gene.

#### Next generation sequencing data analysis

Next generation sequencing analysis of the results of selection experiments was performed as previously described.^37^ Briefly, Illumina sequencing reads were demultiplexed with bcl2fastq2-v2.17.1 and the 21 bp variant sequence was extracted from each read. Variants were counted in each sample and normalized to the sequencing depth of the run to assign each variant a reads per million (RPM) score. Variants were ranked according to the ratio of variant RPM in the sample to variant RPM in the matched sequenced virus library sample to account for unequal distribution of variants in the injected virus library. The highest scoring amino acid variants from the first round of selection in each animal model (10,000 from mice and 20,000 from cynomolgus macaques) were chosen for the second round selection. For each such amino acid variant, a sequence encoding the same peptide by synonymous DNA codons was included in the design of the second-round library to control for DNA sequence-specific effects. For variants with multiple synonymous sequences already observed in experimental samples, the highest scoring synonymous variant was included. For other variants, an artificial sequence was generated by randomizing each codon in the original sequence to a synonymous codon where possible. 5% of variants in the second round library encoded stop codons and were artificially added to the library to control for cross-packaging events during virus library production. Following the second round of selection, DNA sequence variants were ranked as described above, and amino acid variants were ranked according to the sum of the ranks of the two corresponding synonymous sequences. Variants identified in the selection with the fixed PAL motif were ranked as in the first round of selection.

For the pooled rAAV characterization experiment, a ratio was calculated for each barcode of the sample RPM to the RPM of that barcode in the matched sequenced virus library. For each capsid variant, the 10 strongest and 5 weakest barcodes across all samples in the sequencing run were identified according to this metric and removed as outliers from downstream analysis. The remaining 35 midrange barcodes for each variant were then used to determine average transgene expression in each sample as described above.

#### Macaque variant clustering

Pairwise dissimilarity scores between the top 1000 CNS-tropic capsid variants (corrected for synonymous DNA codon sequences) were calculated by adding the single-residue substitution score at each of the seven positions according to the BLOSUM62 substitution matrix. A matrix of dissimilarity scores was converted into a distance matrix by computing the distance metric *d(s, t)* between any two peptide sequences *s* and *t* by analogy with the scalar product as follows:

$$d(s,t)=\sqrt{\langle s|s\rangle +\langle t|t\rangle -2\langle s|t\rangle }$$

where *〈*𝑠|𝑡*〉* is the dissimilarity score between *s* and *t*. This distance matrix was then used for *k*-medoids clustering with the scikit-learn package. The number of clusters *k* was chosen by maximizing the silhouette score.

#### Computational protein modeling

Computational modeling of the VR-VIII loop of MDV1A, MDV1B, PAL1A, and PAL-like.1 was performed on the ProMod3-powered SWISS-MODEL server.^70,71^ AAV9 was used as a template for homology modeling (PDB: 3UX1)^39^ and all structures were visualized in PyMOL.

#### Histology

Whole brains harvested from mice were fixed in 4% PFA for 1 h at room temperature, washed with DPBS, and cryoprotected in 30% sucrose at 4°C overnight. Tissues harvested from macaques injected with PAL2- and AAV9-CBh-hFXN were fixed in 4% PFA overnight at 4°C and washed 3 times with DPBS. Fixed macaque tissues were cryoprotected in 15% sucrose at 4°C overnight and then 30% sucrose at 4°C for up to 3 days. Cryoprotected tissues were then embedded in O.C.T. compound (Sakura Finetek USA) and snap frozen in liquid nitrogen-chilled isopentane. Frozen tissue blocks were sectioned at a thickness of 12 μm on a CM1860 cryostat (Leica Biosystems) and mounted onto Superfrost Plus slides (VWR).

Colorimetric immunohistochemistry experiments (Fig. 2F, S4) were performed with a horseradish peroxidase (HRP) micropolymer kit (Abcam) according to the manufacturer’s instructions except where noted below. Mouse brain cryosections were stained with a rabbit anti-GFP primary antibody (1:1000, Thermo Fisher, A11122) at 4°C overnight in blocking buffer containing 5% normal goat serum (Jackson ImmunoResearch), 2% bovine serum albumin, 2% M.O.M. protein concentrate (Vector Labs), and 0.1% Tween-20. Macaque liver cryosections were first permeabilized in 5% normal goat serum with 0.2% Triton X-100 prior to blocking and staining with rabbit anti-HA (1:1000, Thermo Fisher, MA5-27915) or rabbit anti-FLAG (1:1000, Thermo Fisher, 740001) primary antibodies as above. Following primary antibody incubation, sections were washed three times with PBS and incubated with HRP conjugate at RT for 30 minutes and the signal was visualized with 3,3’-diaminobenzidine (Abcam) prepared according to the manufacturer’s instructions for 3 minutes at RT. Some slides were additionally counterstained with hematoxylin (Abcam) for 30 seconds at 50% concentration. Slides were dehydrated in a graded ethanol series, cleared with three changes of CitriSolv (Decon Laboratories), and mounted with VectaMount (Vector Labs). Sections were imaged on an EVOS M7000 all-in-one microscope using 4X, 20X, and 40X fluorite objective lenses and a color camera with matched power, exposure, and gain across all samples of the same type.

For immunofluorescence experiments (Fig. 7C–H; S2B–F; S3), cryosections of macaque brain, spinal cord, and retina tissue were permeabilized for 10 minutes in 5% normal goat serum with 0.2% Triton X-100 before being blocked at room temperature for 1 hour in blocking buffer containing 5% normal goat serum, 2% bovine serum albumin, 2% M.O.M. protein concentrate, and 0.1% Tween-20. Primary antibody incubations were performed overnight at 4°C in blocking buffer; primary antibodies used were mouse anti-HA (for brain and spinal cord, 1:100, Thermo Fisher, 32–6700), rabbit anti-NeuN (1:250, Abcam, ab177487), rabbit anti-S100β (1:250, Abcam, ab52642), rabbit anti-HA (for retina, 1:500, Thermo Fisher, MA5-27915), and mouse anti-rhodopsin (1:1000, Thermo Fisher, MA1-722). Sections were washed three times with PBS before being incubated at room temperature for 1 hour in blocking buffer with secondary antibodies; secondary antibodies used were goat anti-mouse Alexa Fluor Plus 488 (for brain and spinal cord, 1:500, Thermo Fisher, A32723), goat anti-rabbit Alexa Fluor Plus 594 (for brain and spinal cord, 1:500, Thermo Fisher, A32740), goat anti-rabbit Alexa Fluor Plus 488 (for retina, 1:1000, Thermo Fisher, A32731), and donkey anti-mouse Alexa Fluor Plus 594 (for retina, 1:1000, Thermo Fisher, A32744). Brain and spinal cord sections were mounted with VECTASHIELD antifade mounting media (Vector Labs). Retina sections were treated with the TrueVIEW autofluorescence quenching kit (Vector Labs) according to the manufacturer’s instructions, counterstained with Hoechst 33342 (Thermo Fisher), and mounted with VECTASHIELD Vibrance antifade mounting media (Vector Labs). Tissue sections were imaged on an EVOS M7000 all-in-one microscope using 20X and 40X fluorite objective lenses with matched power, exposure, and gain across samples of the same type. Images of the spinal cord were taken in the ventral horn.

For assessment of spinal cord and DRG pathology (Fig. S5), macaque spinal cord and DRG sections were stained with hematoxylin and eosin (Abcam) according to the usual method. A board-certified neuropathologist who was blinded to the experimental design reviewed anonymized slides and assigned a severity score between 0 (within normal limits) and 5 as previously described.^45^ Severity scores were established for the spinal cord and DRG on sections from three segments each from the cervical, thoracic, and lumbar regions.

#### Quantitative image analysis

All quantitative image processing and analysis was performed with CellProfiler v4.2.^72^ Raw images for each color channel were corrected for variations in background illumination on a per-image basis and segmented into objects using Otsu’s method with filtering for object size. The total number of neurons or astrocytes per image was taken to be the number of objects passing filter in the channels corresponding to NeuN and S100β signal, respectively. Cells were scored as transduced if at least 30% of the area of a cell overlapped with an object passing filter in the channel corresponding to HA signal. The intensities of images selected for display (Fig. 7C–H, S2B–F) were rescaled for ease of visualization.

### Quantification and Statistical Analysis

All statistical analyses were performed in GraphPad Prism v9 (GraphPad Software). All data are presented as mean ± SD where applicable. Datasets were tested for normality using the Shapiro-Wilk test at a significance level of 0.01. All datasets were tested for outliers using the ROUT method and Q = 0.5%. Outliers were identified and removed from AAV9-injected female C57BL/6J spinal cord RNA and DNA (one outlier each, Fig. 2) and C57BL/6J brain and liver RNA (one outlier each where the entire animal was removed from analysis, Fig. 4). For comparisons between AAV9 and MDV1A or PAL2 (Fig. 2D,E; Fig. 7B; Fig. S2A), differences were tested for significance with Welch’s *t*-test with Holm–Šidák correction for multiple comparisons. Quantitative image data (Fig. 7B, S2A) are presented as the mean ± SD of replicate sections, and each data point is the aggregate of three non-overlapping images from the same section. For comparisons between AAV9 and multiple other variants (Fig. 4–6), differences were tested for significance with a one-way ANOVA assuming equal variance and Dunnett’s multiple comparison test using AAV9 as a control mean. All statistical tests were performed on raw data without normalization to AAV9, though mRNA expression data are presented normalized to the mean of AAV9 expression for greater interpretability. Detailed information on statistical analysis for individual figures can be found in the accompanying figure legend.

## Supplementary Material

1

2

3 **Table S1.** Highly enriched sequence and amino acid variants in mice and macaques following two rounds of selection with DELIVER, related to Figures 1–3.
In “Supplemental Videos and Spreadsheets”

## Figures and Tables

**KEY RESOURCES TABLE:** 

REAGENT or RESOURCE	SOURCE	IDENTIFIER
Antibodies		
Rabbit anti-GFP	Thermo Fisher	Cat #A11122; RRID: AB_221569
Rabbit anti-HA	Thermo Fisher	Cat #MA5-27915; RRID: AB_2744968
Rabbit anti-FLAG	Thermo Fisher	Cat #740001; RRID: AB_2610628
Mouse anti-HA	Thermo Fisher	Cat #32-6700; RRID: AB_2533092
Rabbit anti-NeuN	Abcam	Cat #ab177487; RRID: AB_2532109
Rabbit anti-S100b	Abcam	Cat #ab52642; RRID: AB_882426
Mouse anti-rhodopsin	Thermo Fisher	Cat #MA1-722; RRID: AB_325050
Goat anti-mouse Alexa Fluor Plus 488	Thermo Fisher	Cat #A32723; RRID: AB_2633275
Goat anti-rabbit Alexa Fluor Plus 594	Thermo Fisher	Cat #A32740; RRID: AB_2762824
Goat anti-rabbit Alexa Fluor Plus 488	Thermo Fisher	Cat #A32731; RRID: AB_2633280
Donkey anti-mouse Alexa Fluor Plus 594	Thermo Fisher	Cat #A32744; RRID: AB_2762826
Bacterial and Virus Strains		
Endura chemically competent cells	Lucigen	Cat #60240-2
Chemicals, Peptides, and Recombinant Proteins		
RNAlater stabilization solution	Thermo Fisher	Cat #AM7024
DMEM, high glucose, GlutaMAX Supplement, HEPES	Thermo Fisher	Cat #10564029
Fetal bovine serum, certified, One Shot format	Thermo Fisher	Cat #A3160402
PEI MAX	Polysciences	Cat #24765-1
TRIzol reagent	Thermo Fisher	Cat #15596026
QuickExtract DNA extract solution	Lucigen	Cat #QE09050
Tissue-Tek O.C.T. compound	Sakura Finetek	Cat #4583
Superfrost Plus slides	VWR	Cat #48311-703
Normal goat serum	Jackson ImmunoResearch	Cat #005-000-121
Hoechst 33342	Thermo Fisher	Cat #H3570
VECTASHIELD antifade mounting medium	Vector Laboratories	Cat #H-1400-10
VectaMount permanent mounting medium	Vector Laboratories	Cat #H-5000-60
CitriSolv	Decon Labs	Cat #1601
Critical Commercial Assays		
SuperScript IV reverse transcriptase	Thermo Fisher	Cat #18090050
NEBuilder HiFi DNA assembly master mix	New England Biolabs	Cat # E2621
Mouse GAPDH mRNA taqman assay	Integrated DNA Technologies	Cat #Mm.PT.39a.1
Oligo d(T)25 magnetic beads	New England Biolabs	Cat #S1419S
TURBO DNase	Thermo Fisher	Cat #AM2239
Q5 High-Fidelity 2X Master Mix	New England Biolabs	Cat #M0492
HRP/DAB micropolymer IHC detection kit	Abcam	Cat #ab236466
Mouse on Mouse (M.O.M.) immunodetection kit	Vector Laboratories	Cat #BMK-2202
H&E staining kit	Abcam	Cat #ab245880
TrueVIEW autofluorescence quenching kit	Vector Laboratories	Cat #SP-8400-15
Deposited Data		
NGS data	NCBI sequence read archive	Bioproject ID #PRJNA895493
Experimental Models: Cell Lines		
HEK293	ATCC	Cat #CRL-1573
Experimental Models: Organisms/Strains		
Mouse: C57BL/6J	Jackson Laboratory	RRID: IMSR_JAX:000664
Mouse: BALB/cJ	Jackson Laboratory	RRID: IMSR_JAX:000651
Cynomolgus macaque	Biomere Biomedical Research Models	N/A
Oligonucleotides		
Taqman assay primers and probes	See Table S4 for a list of primer and probe sequences	N/A
Recombinant DNA		
Helper plasmid	Aldevron	Cat #pALD-X80
Rep-AAP plasmid	Dr. Benjamin Deverman	N/A
Software and Algorithms		
CellProfiler v4.2	Broad Institute	doi.org/10.1186/s12859-021-04344-9
GraphPad Prism v9	GraphPad Software	https://www.graphpad.com/scientific-software/prism/
NGS analysis code	In house	10.5281/zenodo.7262232

## Highlights

- Directed evolution yields CNS-tropic AAV capsids in mice and juvenile macaques
- PAL family variants are CNS-tropic in macaques after systemic administration
- PAL family variants are de-targeted from the macaque liver
- Mouse-derived variants lack potency in macaques and vice-versa

## Context and Significance

Naturally occurring adeno-associated virus capsids could be used as a strategy for neurological gene therapy, but they are limited by inefficient gene delivery to the brain and blockade of viral particles in the liver. Previous efforts to improve capsids in mice have failed to work in primate models, so researchers at the Broad Institute in Cambridge, Massachusetts used a “directed evolution” strategy in both mice and macaques to identify capsids with better properties. The PAL family of variants identified in macaques lacks potency in mice but shows significantly enhanced ability to reach the brain and reduced off-target effects in the liver in young macaques. These are desirable properties for translational applications in humans and present an opportunity for further refinement.
