# Supplementary material for: Systemic administration of novel engineered AAV capsids facilitates enhanced transgene expression in the macaque CNS
Source: Med. Author manuscript; Available in PMC 2023 Jan 15. (PMC9840684; doi:10.1016/j.medj.2022.11.002)
Supplement: 1 [file NIHMS1852296-supplement-1.docx]

## KEY RESOURCES TABLE

| REAGENT or RESOURCE | SOURCE | IDENTIFIER |
| --- | --- | --- |
| Antibodies | | |
| Rabbit anti-GFP | Thermo Fisher | Cat #A11122; RRID: AB_221569 |
| Rabbit anti-HA | Thermo Fisher | Cat #MA5-27915; RRID: AB_2744968 |
| Rabbit anti-FLAG | Thermo Fisher | Cat #740001; RRID: AB_2610628 |
| Mouse anti-HA | Thermo Fisher | Cat #32-6700; RRID: AB_2533092 |
| Rabbit anti-NeuN | Abcam | Cat #ab177487; RRID: AB_2532109 |
| Rabbit anti-S100b | Abcam | Cat #ab52642; RRID: AB_882426 |
| Mouse anti-rhodopsin | Thermo Fisher | Cat #MA1-722; RRID: AB_325050 |
| Goat anti-mouse Alexa Fluor Plus 488 | Thermo Fisher | Cat #A32723; RRID: AB_2633275 |
| Goat anti-rabbit Alexa Fluor Plus 594 | Thermo Fisher | Cat #A32740; RRID: AB_2762824 |
| Goat anti-rabbit Alexa Fluor Plus 488 | Thermo Fisher | Cat #A32731; RRID: AB_2633280 |
| Donkey anti-mouse Alexa Fluor Plus 594 | Thermo Fisher | Cat #A32744; RRID: AB_2762826 |
| Bacterial and Virus Strains | | |
| Endura chemically competent cells | Lucigen | Cat #60240-2 |
| Chemicals, Peptides, and Recombinant Proteins | | |
| RNAlater stabilization solution | Thermo Fisher | Cat #AM7024 |
| DMEM, high glucose, GlutaMAX Supplement, HEPES | Thermo Fisher | Cat #10564029 |
| Fetal bovine serum, certified, One Shot format | Thermo Fisher | Cat #A3160402 |
| PEI MAX | Polysciences | Cat #24765-1 |
| TRIzol reagent | Thermo Fisher | Cat #15596026 |
| QuickExtract DNA extract solution | Lucigen | Cat #QE09050 |
| Tissue-Tek O.C.T. compound | Sakura Finetek | Cat #4583 |
| Superfrost Plus slides | VWR | Cat #48311-703 |
| Normal goat serum | Jackson ImmunoResearch | Cat #005-000-121 |
| Hoechst 33342 | Thermo Fisher | Cat #H3570 |
| VECTASHIELD antifade mounting medium | Vector Laboratories | Cat #H-1400-10 |
| VectaMount permanent mounting medium | Vector Laboratories | Cat #H-5000-60 |
| CitriSolv | Decon Labs | Cat #1601 |
| Critical Commercial Assays | | |
| SuperScript IV reverse transcriptase | Thermo Fisher | Cat #18090050 |
| NEBuilder HiFi DNA assembly master mix | New England Biolabs | Cat # E2621 |
| Mouse GAPDH mRNA taqman assay | Integrated DNA Technologies | Cat #Mm.PT.39a.1 |
| Oligo d(T)_25_ magnetic beads | New England Biolabs | Cat #S1419S |
| TURBO DNase | Thermo Fisher | Cat #AM2239 |
| Q5 High-Fidelity 2X Master Mix | New England Biolabs | Cat #M0492 |
| HRP/DAB micropolymer IHC detection kit | Abcam | Cat #ab236466 |
| Mouse on Mouse (M.O.M.) immunodetection kit | Vector Laboratories | Cat #BMK-2202 |
| H&E staining kit | Abcam | Cat #ab245880 |
| TrueVIEW autofluorescence quenching kit | Vector Laboratories | Cat #SP-8400-15 |
| Deposited Data | | |
| NGS data | NCBI sequence read archive | Bioproject ID #PRJNA895493 |
| Experimental Models: Cell Lines | | |
| HEK293 | ATCC | Cat #CRL-1573 |
| Experimental Models: Organisms/Strains | | |
| Mouse: C57BL/6J | Jackson Laboratory | RRID: IMSR_JAX:000664 |
| Mouse: BALB/cJ | Jackson Laboratory | RRID: IMSR_JAX:000651 |
| Cynomolgus macaque | Biomere Biomedical Research Models | N/A |
| Oligonucleotides | | |
| Taqman assay primers and probes | See Table S4 for a list of primer and probe sequences | N/A |
| Recombinant DNA | | |
| Helper plasmid | Aldevron | Cat #pALD-X80 |
| Rep-AAP plasmid | Dr. Benjamin Deverman | N/A |
| Software and Algorithms | | |
| CellProfiler v4.2 | Broad Institute | doi.org/10.1186/s12859-021-04344-9 |
| GraphPad Prism v9 | GraphPad Software | https://www.graphpad.com/scientific-software/prism/ |
| NGS analysis code | In house | 10.5281/zenodo.7262232 |
