# Supplementary material for: Systemic administration of novel engineered AAV capsids facilitates enhanced transgene expression in the macaque CNS
Source: Med. Author manuscript; Available in PMC 2023 Jan 15. (PMC9840684; doi:10.1016/j.medj.2022.11.002)
Supplement: 2 [file NIHMS1852296-supplement-2.pdf]

A Cerebellum

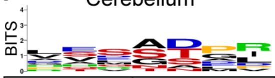

Rank	7-mer Sequence
1	LESAAMI
2	PAQGTLR
3	VHDSTPL
4	LSMTDGL
5	GVVNDER
6	HTLSTGV
7	DVRGSDI
8	ASESSPP
9	VQSADPR
10	RESANAD

B Spinal Cord

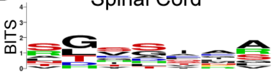

Rank	7-mer Sequence
1	STVGINV
2	VGSLTAS
3	TGSSAML
4	NGYTEGR
5	RDEAYRA
6	ALGDQAR
7	SGISFLA
8	STTSSPS
9	LLHGIIA
10	RHVHVEG

C

Cluster A

Sequence	Rank
SHGSDPK	12
SHGYDSK	19
DHGSFAK	122
SHGTDSK	127
SHGSDLK	145
YHASDSK	176
SHGSDSN	275
SHGSDSR	316
FHGSDSK	525
SHGTDTK	575
SHASDSK	695
NFGRDTL	710
SHGSDTK	963

Cluster B

Sequence	Rank
MNVGHVL	24
MNGGNVL	314
LNGAPLL	326
MNGGHLM	365
MIGGHVQ	380
MNGGHVL	393
VSNGHFV	440
KNGGHDL	483
MNGGHVQ	689
MNGGHVI	732
MNGGHVM	779

Cluster C

Sequence	Rank
PNERLAV	202
PNERHTL	318
PNTRDPI	418
PNDRLTV	449
PNERLTQ	526
PNERLTL	794
HQDRTTL	958

- nonpolar: G, A, V, I, L, M, F, W
- polar: S, T, N, Q, C, Y
- acidic: D, E
- basic: R, H, K
- proline (P)

Figure S1. Selection for capsid variants with CNS-tropic properties in cynomolgus macaques yields diverse families of motifs, related to Figure 3.
(A and B) Amino acid sequence and logo of the 7-mer insert in the 10 most enriched capsid variants in the **(A)** cerebellum and **(B)** spinal cord of cynomolgus macaques injected with 3E+13 vg/kg virus library following two rounds of selection with DELIVER. The rank of each variant corresponds to the sum of the ranks of two synonymous DNA sequences.
(C) Selected clusters of enriched variants in the macaque CNS with conserved sequence properties. Individual residues are color-coded according to their functional properties to highlight conserved aspects of the sequence motif. The rank of each variant corresponds to the sum of the ranks of two synonymous DNA sequences.

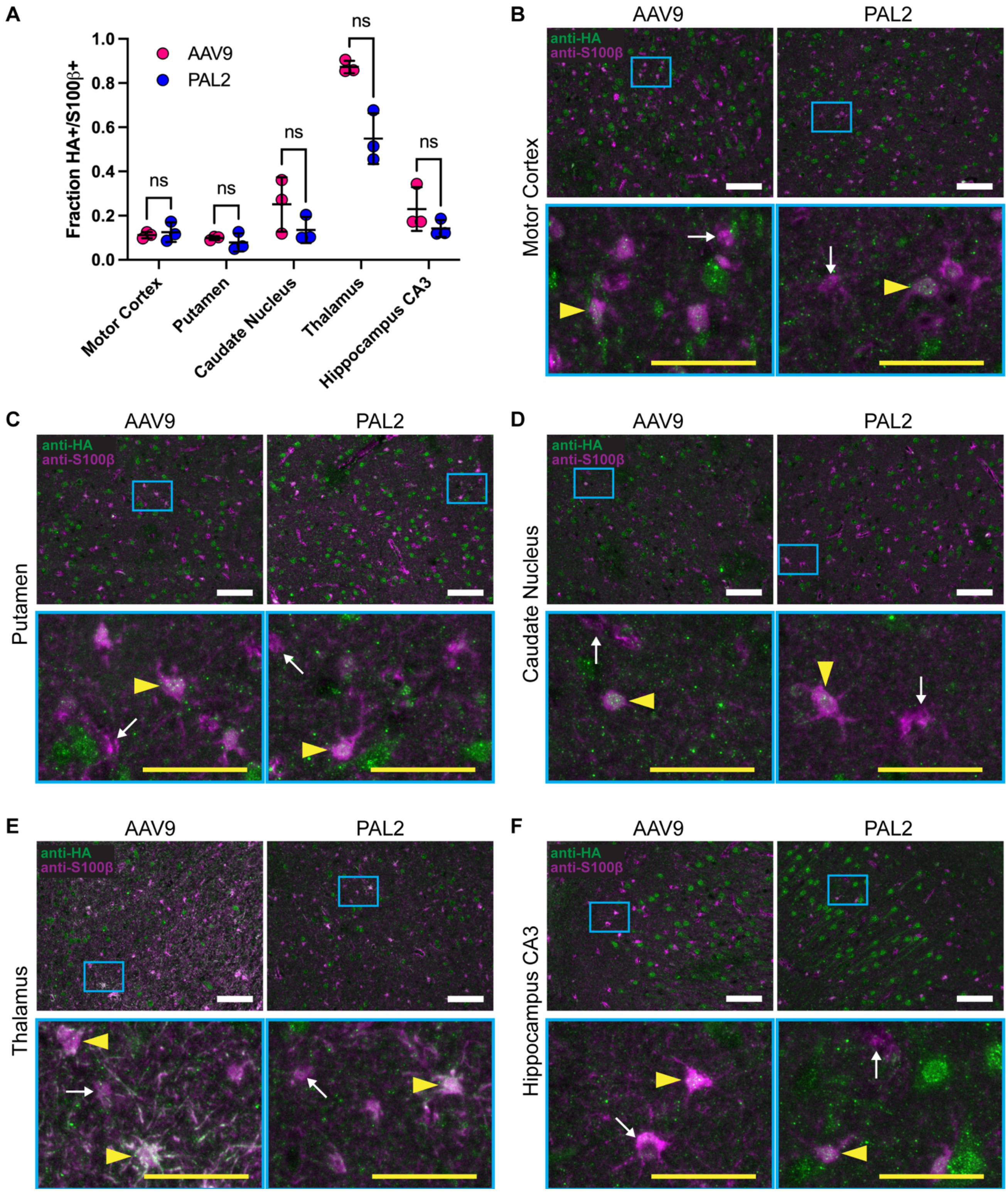
A
Fraction HA+/S100β+
1.0
0.8
0.6
0.4
0.2
0.0
AAV9
PAL2
ns
Motor Cortex
Putamen
Caudate Nucleus
Thalamus
Hippocampus CA3
B
C
D
E
F
anti-HA
anti-S100β
Hippocampus CA3

Figure S2. PAL2 and AAV9 transduce similar proportions of S100β+ astrocytes in macaques, related to Figure 7.
(A) Fraction of S100β+ astrocytes transduced by AAV9 or PAL2 delivering a CBh-hFXN-HA transgene as measured by immunostaining for the HA tag in conjunction with S100β. In order to compare the same tag for both vectors, AAV9 and PAL2 data are derived from different two year old cynomolgus macaques injected with 3E+13 vg/kg of the vector in question; one male and one female, respectively. Data are represented as mean ± SD for n=3 replicate tissue sections and individual data points are the aggregate of three non-overlapping images of the same section taken with a 20X objective. Significance is determined by two-tailed Welch's *t*-test with Holm-Šídák MCT.
(B-F) Representative images depicting colocalization of hFXN-HA expression with respect to S100β+ astrocytes in 12 µm cryosections of the macaque **(B)** motor cortex, **(C)** putamen, **(D)** caudate nucleus, **(E)** thalamus, and **(F)** CA3 region of the hippocampus. Magnified frames (cyan) show detailed regions with representative HA+ transduced astrocytes (yellow arrowheads) and HA- untransduced astrocytes (white arrows). White scale bars: 100 µm; yellow scale bars: 50 µm.

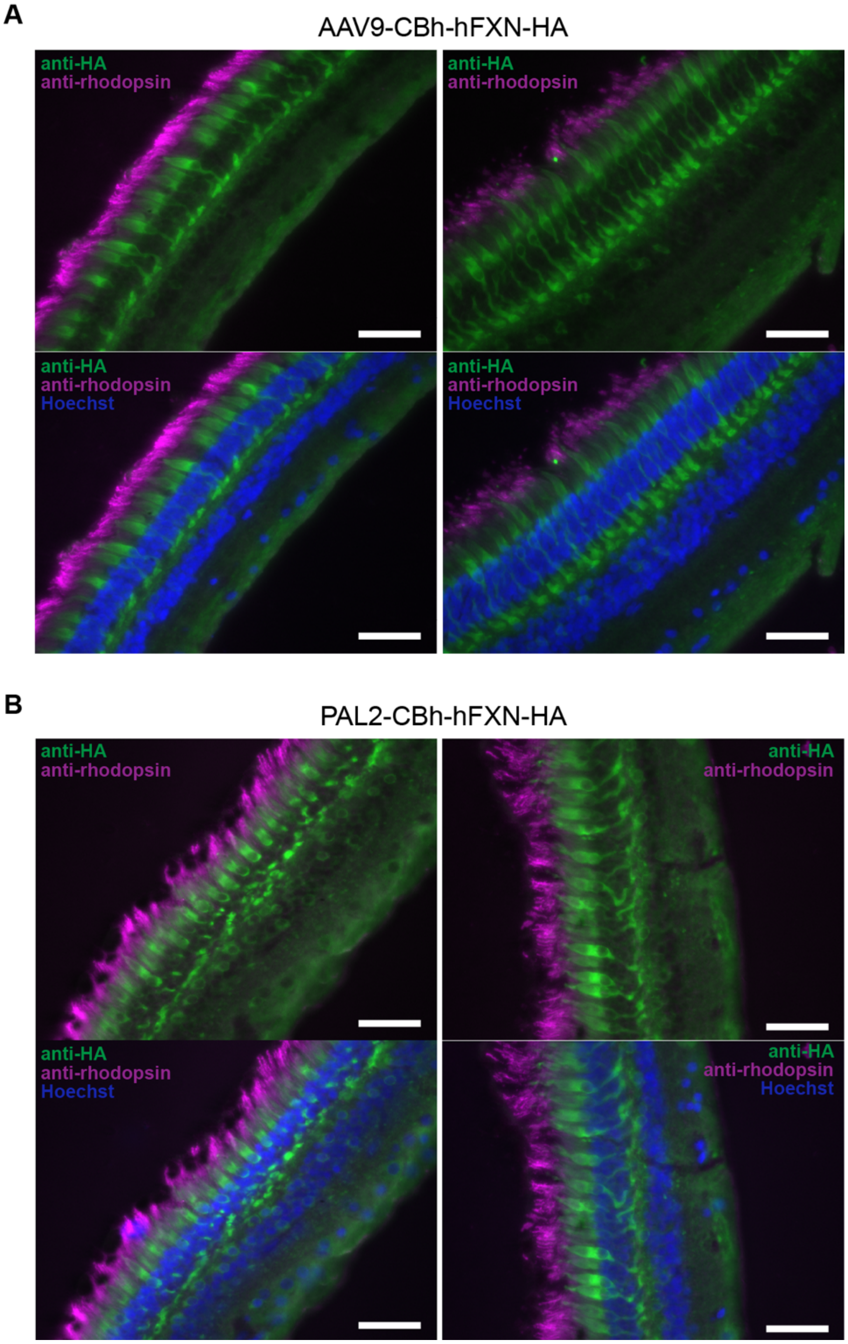

Figure S3. PAL2 and AAV9 transduce photoreceptor cells in the macaque neuroretina, related to Figure 7.

Representative images of 12 μm cryosections of the cynomolgus macaque neuroretina following systemic administration of **(A)** AAV9- or **(B)** PAL2-CBh-hFXN-HA immunostained for the HA tag and rhodopsin. Note high hFXN-HA levels in photoreceptor cells as well as in the outer plexiform layer, located between the inner and outer nuclear layers identifiable by the high density of Hoechst-stained nuclei. Scale bars: 50 μm.

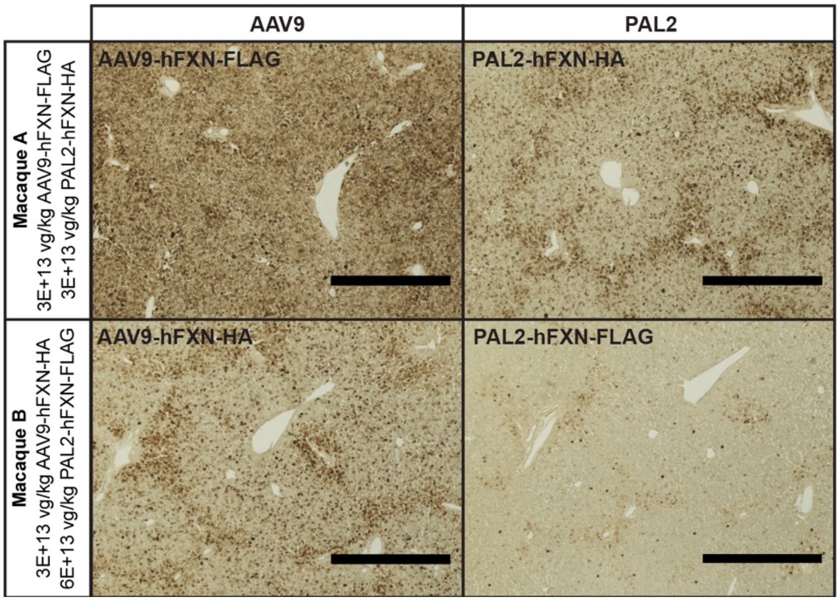

Figure S4. PAL2 transduces fewer cells in the macaque liver compared to AAV9, related to Figure 7. Representative images of the cynomolgus macaque liver following administration of an rAAV cocktail containing AAV- and PAL2-CBh-hFXN immunostained for the HA or FLAG tags. Dose per vector is listed to the left. Scale bars: 100 µm.

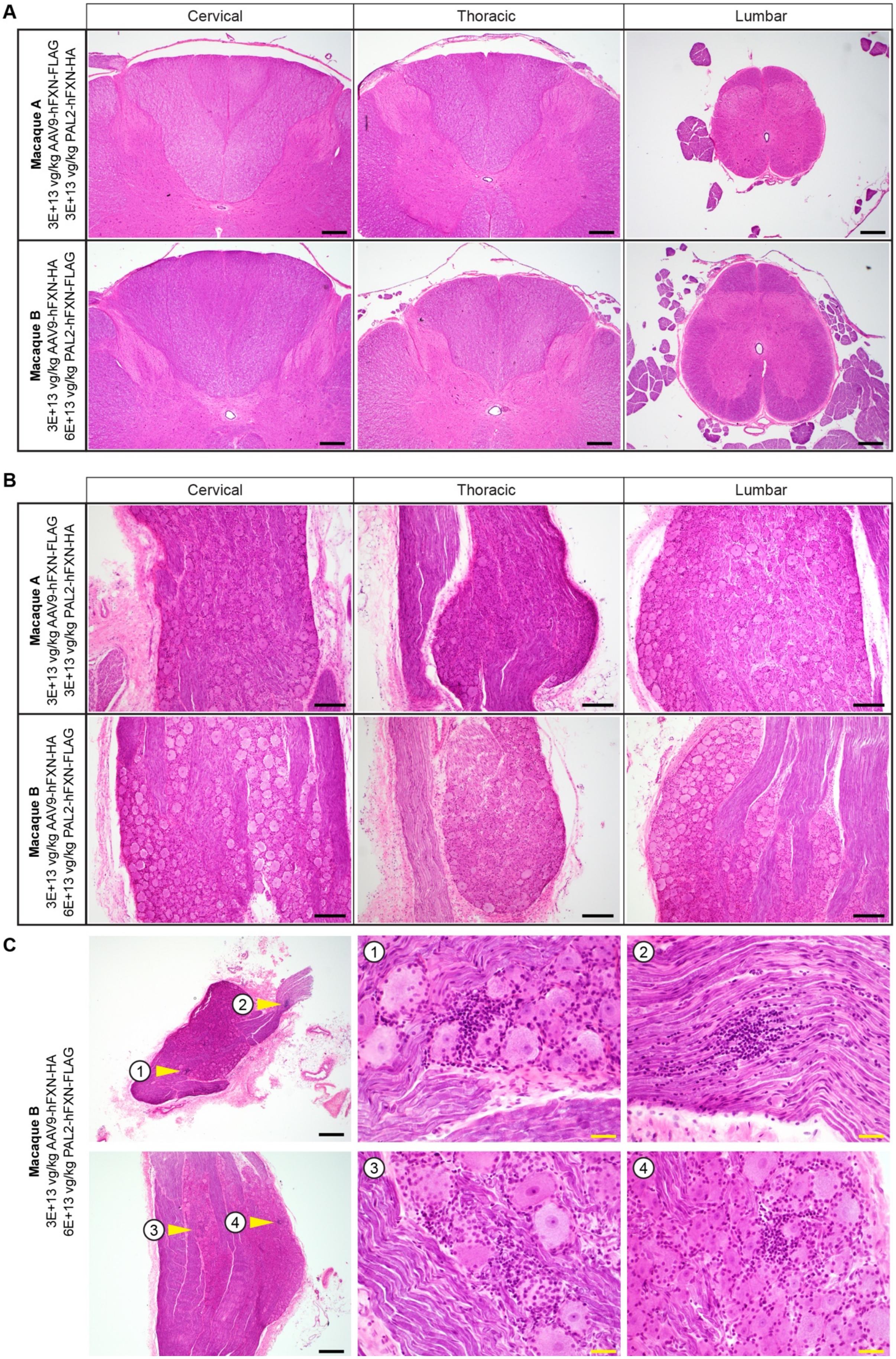
A
Cervical
Thoracic
Lumbar
Macaque A
3E+13 vg/kg AAV9-hFXN-FLAG
3E+13 vg/kg PAL2-hFXN-HA
Macaque B
3E+13 vg/kg AAV9-hFXN-HA
6E+13 vg/kg PAL2-hFXN-FLAG
B
Cervical
Thoracic
Lumbar
Macaque A
3E+13 vg/kg AAV9-hFXN-FLAG
3E+13 vg/kg PAL2-hFXN-HA
Macaque B
3E+13 vg/kg AAV9-hFXN-HA
6E+13 vg/kg PAL2-hFXN-FLAG
C
Macaque B
3E+13 vg/kg AAV9-hFXN-HA
6E+13 vg/kg PAL2-hFXN-FLAG
1
2
3
4

Figure S5. Macaques show no abnormal spinal cord pathology following systemic administration of an rAAV cocktail containing AAV9 and PAL2, while a higher dose results in minor lesions of the DRG, related to Figure 7.
(A) Representative 12 μm cryosections of the cervical, thoracic, and lumbar spinal cord of two macaques injected with a AAV9- and PAL2-CBh-hFXN cocktail with pathology WNL (within normal limits). Dose per vector is listed to the left. Scale bars: 200 μm.
(B) Representative 12 μm cryosections of the cervical, thoracic, and lumbar DRG of two macaques injected with a AAV9- and PAL2-CBh-hFXN cocktail with pathology WNL. Scale bars: 100 μm.
(C) Detail of lesions in two grade 1/5 lumbar DRG of the macaque injected with the higher dose rAAV cocktail. Numbered yellow arrows indicate lesions presented at higher magnification to the right. Black scale bars: 200 μm; yellow scale bars: 20 μm.

Table S1. Highly enriched sequence and amino acid variants in mice and macaques following two rounds of selection with DELIVER, related to Figures 1-3.

In "Supplemental Videos and Spreadsheets"

Table S2. PAL2 and AAV9 transgene expression and vector genome abundance in macaque A, related to Figure 7.

Macaque A: 2 year old female injected with 3E+13 vg/kg each AAV9-hFXN-FLAG and PAL2-hFXN-HA.

Tissue	PAL2 mRNA[a]	AAV9 mRNA[a]	PAL2/AAV9 mRNA	PAL2 vector genomes/cell[b]	AAV9 vector genomes/cell[b]	PAL2/AAV9 vector DNA
Frontal lobe	3.81E-05	7.82E-06	4.87	6.25E-03	4.86E-03	1.29
Temporal lobe (anterior)	4.80E-05	1.10E-05	4.37	1.13E-02	1.09E-02	1.04
Temporal lobe (posterior)	6.27E-05	1.17E-05	5.35	1.01E-02	8.07E-03	1.25
Parietal lobe (anterior)	7.98E-05	1.35E-05	5.93	1.10E-02	9.11E-03	1.21
Parietal lobe (posterior)	1.19E-04	2.28E-05	5.23	1.37E-02	1.13E-02	1.22
Occipital lobe	1.06E-04	1.93E-05	5.50	6.73E-03	4.83E-03	1.39
Thalamus	5.54E-05	1.20E-05	4.61	2.04E-02	7.96E-03	2.56
Midbrain	9.67E-05	1.97E-05	4.90	9.28E-03	5.01E-03	1.85
Corpus callosum	8.27E-05	3.03E-05	2.73	3.67E-03	1.93E-03	1.91
Cerebellum	6.69E-05	3.16E-05	2.11	2.18E-03	1.44E-03	1.51
Neuroretina	1.63E-04	1.22E-05	13.40	3.19E-03	8.52E-04	3.75
RPE	4.01E-04	1.72E-04	2.34	1.12E-01	1.85E-01	0.61
Brain stem	9.34E-05	4.00E-05	2.33	8.97E-03	5.76E-03	1.56
Cervical spinal cord	4.34E-04	1.44E-04	3.01	2.08E-02	1.42E-02	1.46
Thoracic spinal cord	9.40E-04	3.19E-04	2.94	2.38E-02	1.84E-02	1.29
Lumbar spinal cord	1.71E-03	4.78E-04	3.58	3.15E-02	1.74E-02	1.80
Cauda equina	3.90E-02	6.81E-03	5.74	6.35E-02	3.42E-02	1.86
Cervical DRG	3.12E-02	3.23E-03	9.64	5.08E-02	3.32E-02	1.53
Thoracic DRG	1.55E-02	1.97E-03	7.83	3.57E-02	1.93E-02	1.84
Lumbar DRG	3.98E-02	6.07E-03	6.56	1.74E-01	1.37E-01	1.27
Triceps	5.08E-02	1.72E-02	2.95	2.03E-01	2.49E-01	0.82
Quadriceps	5.37E-03	1.46E-03	3.68	2.70E-02	4.36E-02	0.62
Diaphragm	2.79E-02	8.01E-03	3.48	1.55E-01	1.97E-01	0.79
Heart	2.76E-02	1.15E-02	2.39	1.37E-01	2.04E-01	0.67
Kidney	4.81E-04	1.71E-04	2.81	1.53E-01	1.73E-01	0.88
Lung	8.12E-04	3.95E-04	2.06	3.44E-01	4.10E-01	0.84
Thymus	3.38E-03	1.85E-03	1.83	1.44E-02	4.69E-03	3.06
Gonad	3.20E-03	2.89E-03	1.11	1.83E-02	2.25E-02	0.81
Liver	8.35E-02	1.73E-01	0.48	1.31E+01	5.02E+01	0.26
Spleen	1.84E-04	3.78E-04	0.49	6.29E+00	1.27E+00	4.97

[a]Transgene mRNA expression normalized to expression of GAPDH mRNA as detected by qPCR with a standard curve
[b]Vector DNA normalized to the number of GAPDH genomic DNA copies as detected by qPCR with a standard curve

Table S3. PAL2 and AAV9 transgene expression and vector genome abundance in macaque B, related to Figure 7.

Macaque B: 2 year old male injected with 3E+13 vg/kg AAV9-hFXN-HA and 6E+13 vg/kg PAL2-hFXN-FLAG.

Tissue	PAL2 mRNA[a]	AAV9 mRNA[a]	PAL2/AAV9 mRNA	PAL2 vector genomes/cell[b]	AAV9 vector genomes/cell[b]	PAL2/AAV9 vector DNA
Frontal lobe	1.83E-04	1.31E-05	13.89	1.21E-02	4.62E-03	2.62
Temporal lobe (anterior)	1.45E-04	1.14E-05	12.71	1.73E-02	6.79E-03	2.55
Temporal lobe (posterior)	2.17E-04	1.39E-05	15.57	1.63E-02	4.68E-03	3.49
Parietal lobe (anterior)	6.13E-04	3.63E-05	16.89	2.39E-02	7.41E-03	3.22
Parietal lobe (posterior)	9.35E-04	4.41E-05	21.19	1.41E-02	4.92E-03	2.87
Occipital lobe	3.45E-04	2.25E-05	15.31	7.24E-03	4.13E-03	1.76
Thalamus	1.38E-04	2.31E-05	6.00	2.01E-02	9.96E-03	2.02
Midbrain	3.20E-04	1.71E-05	18.79	1.79E-02	6.47E-03	2.76
Corpus callosum	1.86E-04	1.86E-05	9.99	6.09E-03	2.13E-03	2.85
Cerebellum	2.07E-04	2.69E-05	7.69	2.49E-03	8.49E-04	2.93
Neuroretina	3.21E-04	1.02E-05	31.56	3.53E-03	1.20E-03	2.95
RPE	4.71E-04	8.38E-05	5.62	6.54E-02	5.36E-02	1.22
Brain stem	4.69E-04	7.95E-05	5.89	7.49E-03	2.50E-03	2.99
Cervical spinal cord	1.16E-03	2.96E-04	3.92	1.75E-02	1.27E-02	1.38
Thoracic spinal cord	7.59E-04	2.87E-04	2.64	2.24E-02	5.23E-02	0.43
Lumbar spinal cord	2.22E-03	5.26E-04	4.21	3.41E-02	1.67E-02	2.04
Cauda equina	3.97E-02	1.41E-02	2.81	7.26E-02	9.94E-02	0.73
Cervical DRG	2.25E-02	7.72E-03	2.91	3.85E-02	6.96E-02	0.55
Thoracic DRG	9.54E-03	4.75E-03	2.01	2.95E-02	3.73E-02	0.79
Lumbar DRG	4.03E-02	1.90E-02	2.12	6.43E-02	6.09E-02	1.06
Triceps	3.64E-02	1.75E-02	2.08	2.39E-01	1.46E-01	1.64
Quadriceps	1.36E-02	6.58E-03	2.06	2.02E-01	1.27E-01	1.59
Diaphragm	4.86E-02	2.15E-02	2.26	2.51E-01	1.23E-01	2.05
Heart	4.03E-02	5.21E-02	0.77	3.22E-01	2.36E-01	1.36
Kidney	1.88E-04	1.23E-04	1.53	6.74E-02	7.44E-02	0.91
Lung	2.00E-03	2.78E-03	0.72	2.51E-01	2.38E-01	1.06
Thymus	3.15E-03	5.09E-03	0.62	6.73E-03	2.94E-03	2.29
Gonad	3.63E-03	7.34E-03	0.49	6.10E-02	7.56E-02	0.81
Liver	1.15E-02	8.23E-02	0.14	9.46E+00	2.70E+01	0.35
Spleen	1.89E-04	1.66E-03	0.11	6.77E+00	5.98E-01	11.32

[a]Transgene mRNA expression normalized to expression of GAPDH mRNA as detected by qPCR with a standard curve

[b]Vector DNA normalized to the number of GAPDH genomic DNA copies as detected by qPCR with a standard curve

Table S4. Taqman assay primers and probes, related to STAR Methods.

**Mouse GAPDH DNA**	
Mouse GAPDH DNA F	5'- GATGTCCTTGGTGCACACTC - 3'
Mouse GAPDH DNA R	5'- GAGCTGAGATTGCCCCGC - 3'
Mouse GAPDH DNA probe (FAM)	5'- CCTCGTCCTTAAGTTCATAGTCTGTATTC - 3'
**EGFP**	
EGFP F	5'- TGTAGTTGCCGTCGTCCTTG - 3'
EGFP R	5'- GCTACCCCGACCACATGAAG - 3'
EGFP probe (HEX)	5'- TCTTCAAGTCCGCCATGCCCG - 3'
**Cynomolgus macaque GAPDH DNA**	
Macaque GAPDH DNA F	5'- GGTGCAGTTTGAAGCTGAGC - 3'
Macaque GAPDH DNA R	5'- CAGTCTGGGCACAAGCTTTG - 3'
Macaque GAPDH DNA probe (FAM)	5'- TGGCATAGTGAGGTGCTGAATGC - 3'
**Cynomolgus macaque GAPDH mRNA**	
Macaque GAPDH mRNA F	5'- ACAACAGCCTCAAGATCGTCAG - 3'
Macaque GAPDH mRNA R	5'- ACTGTGGTCATGAGTCCTTCC - 3'
Macaque GAPDH mRNA probe (FAM)	5'- TCCTGCACCACCAACTGCTTAG - 3'
**hFXN-HA**	
hFXN-HA F	5'- ACCAAACTGGACTTGTCTTCCTTG - 3'
hFXN-HA R	5'- TCAATGTATCTTATCATGTCTGCTCG - 3'
hFXN-HA probe (FAM)	5'- TACCCATACGATGTTCCAGATTACGCT - 3'
**hFXN-FLAG**	
hFXN-FLAG F	5'- ACCAAACTGGACTTGTCTTCCTTG - 3'
hFXN-FLAG R	5'- TCAATGTATCTTATCATGTCTGCTCG - 3'
hFXN-FLAG probe (FAM)	5' - CGATTACAAGGATGACGATGACAAGT - 3'
**AAV9 capsid**	
AAV9 capsid F	5'- CCTCGGGAAATTGGCATTGC - 3'
AAV9 capsid R	5'- ATTGTTGTAGGTGGGCAGGG - 3'
AAV9 capsid probe (HEX)	5'- ACAGAGTCATCACCACCAGCACCCG - 3'
